# Supplementary material for: Genome-wide meta-analysis of myasthenia gravis uncovers new loci and provides insights into polygenic prediction
Source: Nat Commun. 2024 Nov 13;15:9839. doi: 10.1038/s41467-024-53595-6 (PMC11560923; doi:10.1038/s41467-024-53595-6)
Supplement: Supplementary file 1 — Supplementary Information [file 41467_2024_53595_MOESM1_ESM.pdf]

Supplementary online material

Genome-wide meta-analysis of myasthenia gravis uncovers new loci and provides insights into polygenic prediction

Full list of consortia contributors **1**
23andMe, Inc. 1
Estonian Biobank Research Team 1
Supplementary figures **2**
Supplementary Figure 1. Quantile-quantile plots 2
Supplementary Figure 2. Region plots of MG discovery GWAS 3
Supplementary Figure 3. Forest plots of index SNPs for MG 6
Supplementary Figure 4. Forest plots of index SNPs across AChR and mixed MG samples 12
Supplementary Figure 5. Region plots of EOMG discovery GWAS 16
Supplementary Figure 6. Forest plots of index SNPs for EOMG 17
Supplementary Figure 7. Region plots of LOMG discovery GWAS 18
Supplementary Figure 8. Forest plots of index SNPs for LOMG 19
Supplementary Figure 9. Region plots of imputed HLA types in the combined GWAS 21
Supplementary Figure 10. Region plots of imputed HLA types in the early-onset GWAS 22
Supplementary Figure 11. Region plots of imputed HLA types in the late-onset GWAS 23
Supplementary Figure 12. Manhattan plot of TWAS results for skeletal muscle tissue 24
Supplementary Figure 13. Manhattan plot of TWAS results for tibial nerve tissue 24
Supplementary Figure 14. Manhattan plot of TWAS results for whole blood tissue 25
Supplementary Figure 15. Performance of MG PRS across onset phenotypes 26
Supplementary Figure 16. Performance of MG PRS across antibody phenotypes 26
Supplementary Figure 17. Genetic correlation results of pre-selected traits 27
Supplementary Figure 18. Variant lookup in UK Biobank, GWAS Catalog and FinnGen 28
Sample description of genotypes **34**
Charité Universitätsmedizin Berlin 34
Leiden University Medical Center 34
Gregersen et al. 2012 35
Seldin et al. 2015 35
Renton et al. 2015 35
Sample description of summary-level data **36**
deCODE Genetics/ Amgen, Inc. 36
Estonian Biobank 37
Million Veteran Program 38
Supplementary references **40**

Full list of consortia contributors

23andMe, Inc.

We would like to thank the research participants and employees of 23andMe for making this work possible. The following members of the 23andMe Research Team contributed to this study: Stella Aslibekyan, Adam Auton, Elizabeth Babalola, Robert K. Bell, Jessica Bielenberg, Jonathan Bowes, Katarzyna Bryc, Ninad S. Chaudhary, Daniella Coker, Sayantan Das, Emily DelloRusso, Sarah L. Elson, Nicholas Eriksson, Teresa Filshtein, Pierre Fontanillas, Will Freyman, Zach Fuller, Chris German, Julie M. Granka, Karl Heilbron, Alejandro Hernandez, Barry Hicks, David A. Hinds, Ethan M. Jewett, Yunxuan Jiang, Katelyn Kukar, Alan Kwong, Yanyu Liang, Keng-Han Lin, Bianca A. Llamas, Matthew H. McIntyre, Steven J. Micheletti, Meghan E. Moreno, Priyanka Nandakumar, Dominique T. Nguyen, Jared O'Connell, Aaron A. Petrakovitz, G. David Poznik, Alexandra Reynoso, Shubham Saini, Morgan Schumacher, Leah Selcer, Anjali J. Shastri, Janie F. Shelton, Jingchunzi Shi, Suyash Shringarpure, Qiaojuan Jane Su, Susana A. Tat, Vinh Tran, Joyce Y. Tung, Xin Wang, Wei Wang, Catherine H. Weldon, Peter Wilton, Corinna D. Wong.
Affiliation of consortia members: 23andMe, Inc., Sunnyvale, California, United States of America

Estonian Biobank Research Team

The following members of the Estonian Biobank Research Team contributed to this study:
Andres Metspalu, Mari Nelis, Lili Milani, Reedik Mägi, Georgi Hudjashov, Tõnu Esko
Affiliation of consortia members: Institute of Genomics, University of Tartu, Tartu, Estonia

Supplementary figures

Supplementary Figure 1. Quantile-quantile plots

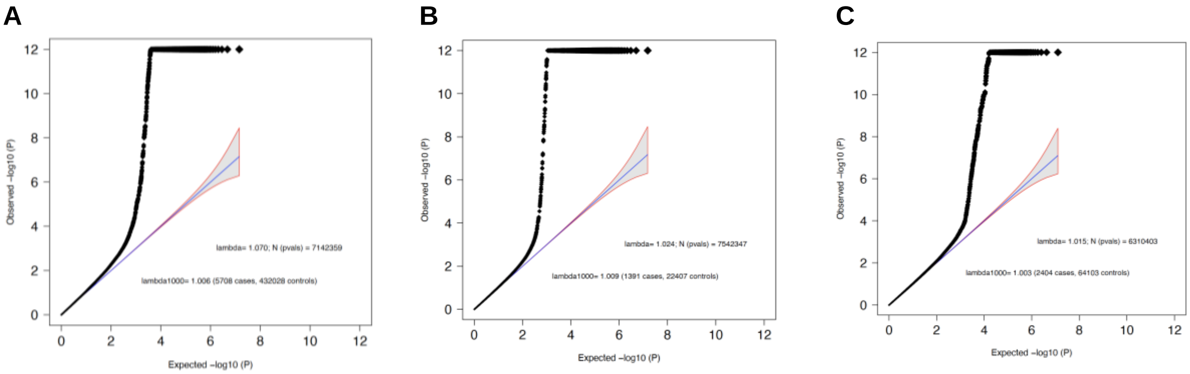

Quantile-quantile plots depicting the expected and observed p-values along with the genomic inflation factor (λGC) of **A** the combined GWAS of MG, **B** the early-onset GWAS, and **C** the late-onset GWAS

Supplementary Figure 2. Region plots of MG discovery GWAS

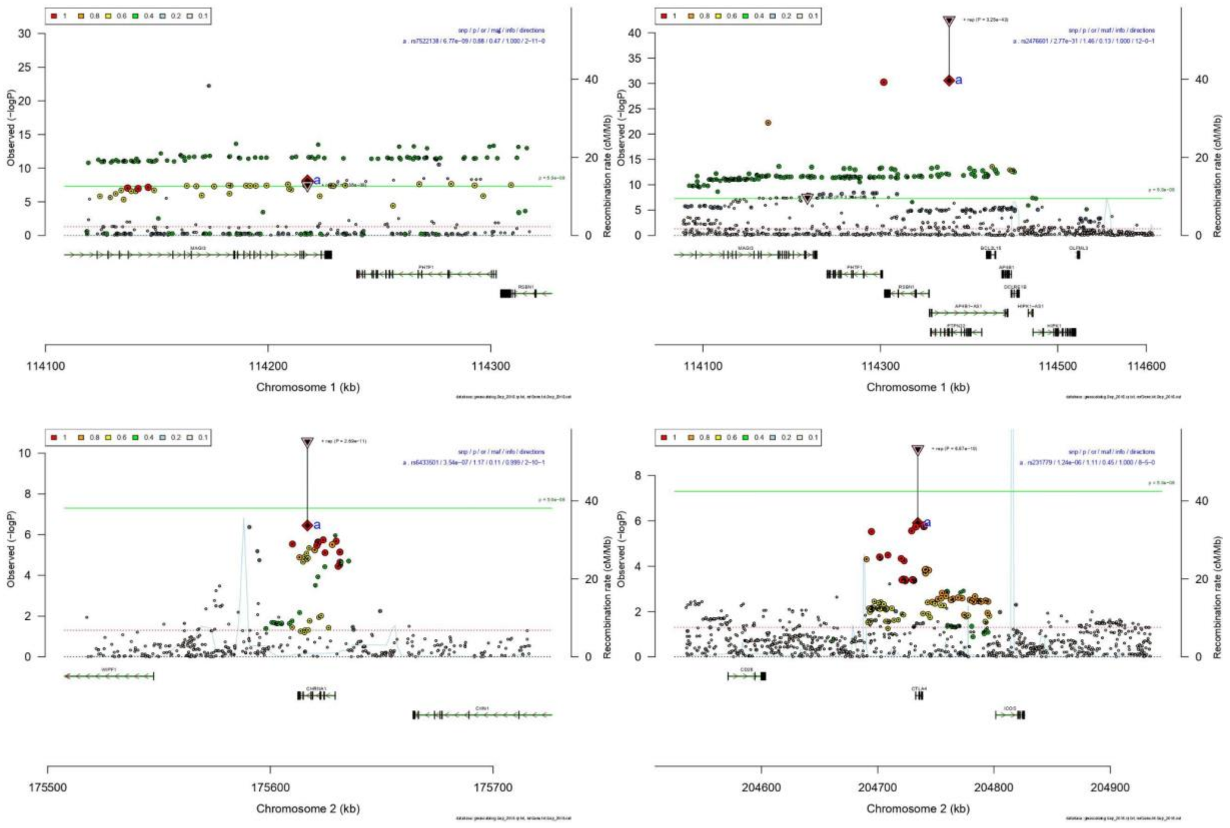

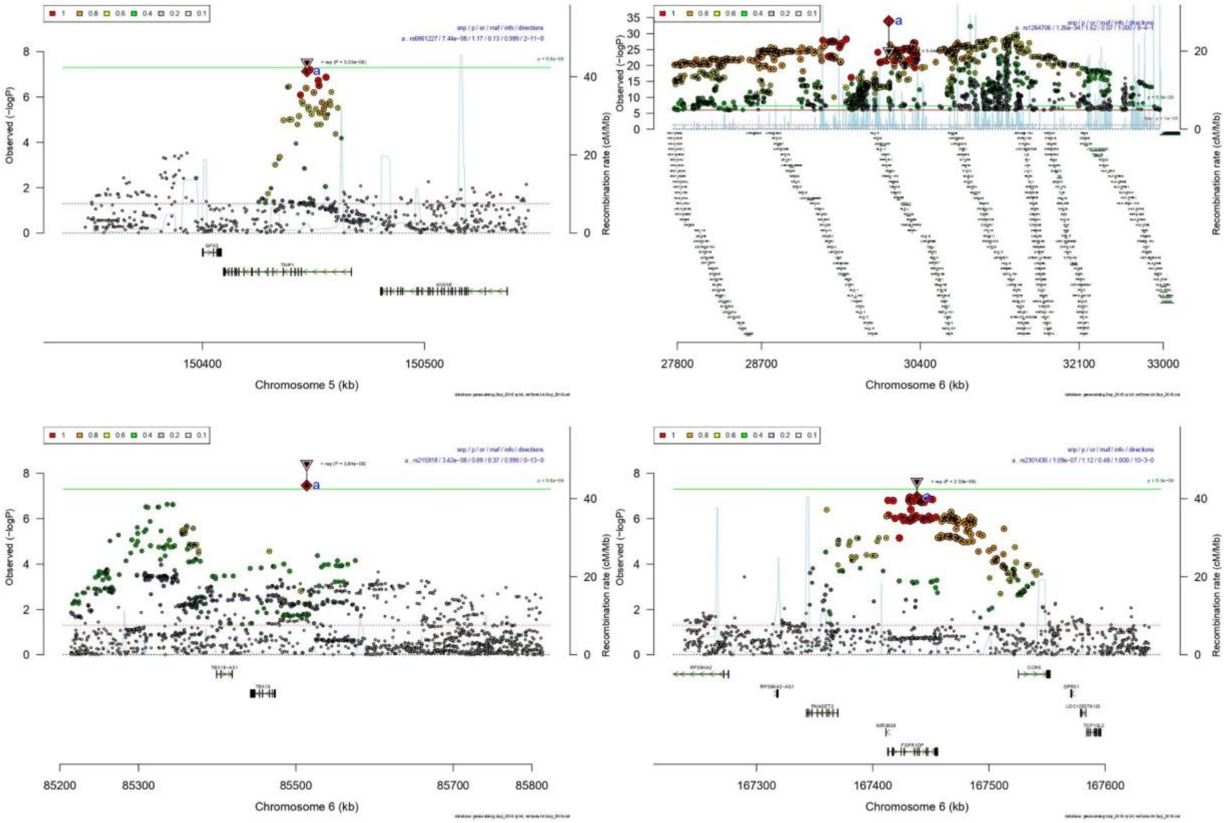

Observed (−logP)
Recombination rate (cM/Mb)
snp / p / or / maf / info / directions
150400
150500
Chromosome 5 (kb)
27800
28700
30400
32100
33000
Chromosome 6 (kb)
85200
85300
85500
85700
85800
Chromosome 6 (kb)
167300
167400
167500
167600
Chromosome 6 (kb)

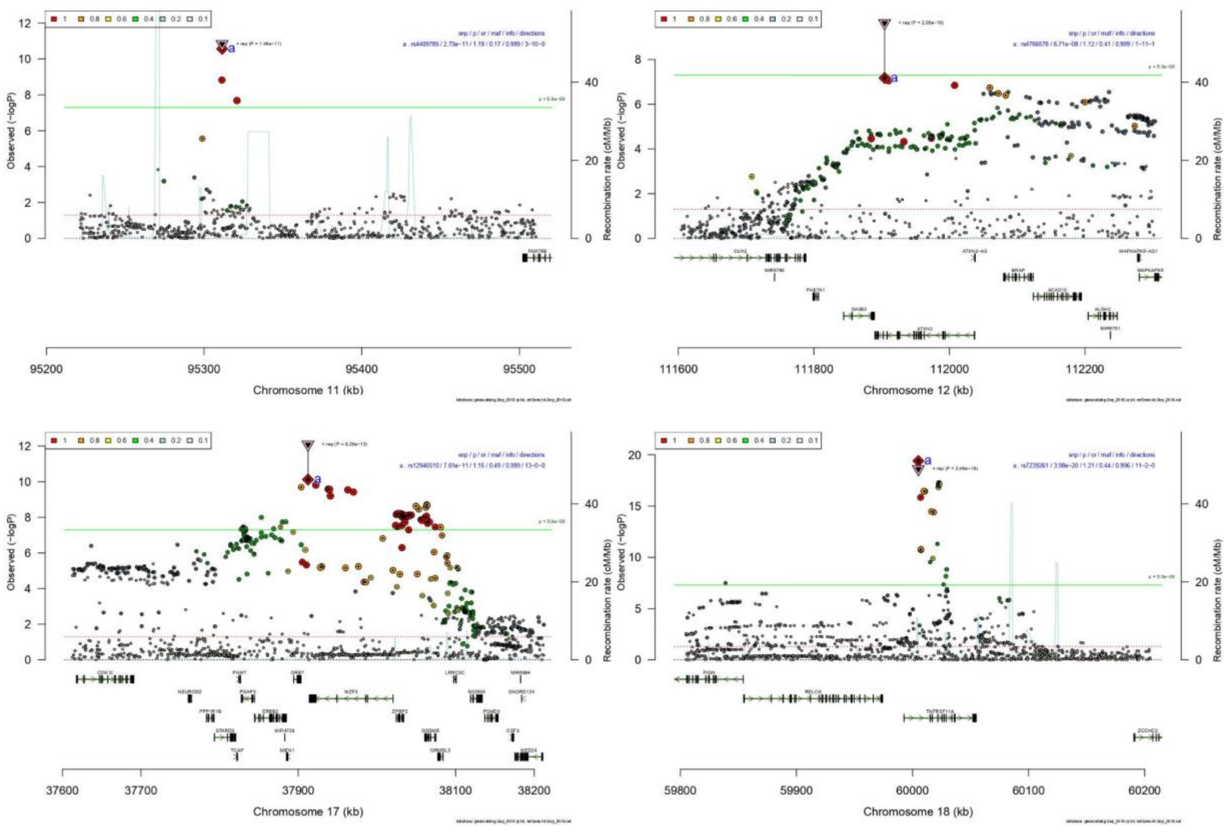

Each point in the plot represents a SNP colored according to the level of linkage disequilibrium with the index SNP. The left x-axis depicts the genomic position and the y-axis indicates the observed -log10 p-value. The right y-axis shows the Recombination rate (cM/Mb). The green line indicates the genome-wide significance threshold of $p<5e\text{-}8$. Triangles indicate the index SNPs p-value in the discovery and replication meta-analysis.

Supplementary Figure 3. Forest plots of index SNPs for MG

CHR1:114217705
rs7522138: A/G

Cohort	N cases	ln(OR)	[95% CI]	p-value
**Discovery**				
FinnGen	329	-0.01	[-0.16; 0.15]	9.4e-01
BioVU	230	-0.09	[-0.30; 0.11]	3.8e-01
Gregersen et al. 2012 mid EU	136	0.20	[-0.08; 0.48]	1.7e-01
Charite LUMC	390	0.13	[-0.05; 0.31]	1.5e-01
Topaloudi et al 2022	196	0.17	[-0.06; 0.39]	1.5e-01
DeCODE Genetics	133	0.23	[-0.03; 0.49]	8.2e-02
Gregersen et al. 2012 ENG	276	0.18	[-0.01; 0.37]	6.6e-02
Million Veterans Program	1,334	0.09	[0.01; 0.18]	3.3e-02
Gregersen et al. 2012 north EU	228	0.26	[0.04; 0.47]	2.0e-02
UK Biobank	288	0.24	[0.05; 0.43]	1.5e-02
Chia et al. 2022	1,873	0.10	[0.03; 0.17]	7.2e-03
Seldin et al. 2015	89	0.48	[0.14; 0.82]	5.1e-03
Estonian Biobank	206	0.36	[0.14; 0.58]	1.3e-03
Discovery	5,708	0.12	[0.08; 0.16]	6.8e-09
**Replication**				
23andMe Replication	3,989	0.04	[-0.00; 0.09]	5.8e-02
Discovery & Replication	9,697	0.09	[0.06; 0.12]	3.4e-08

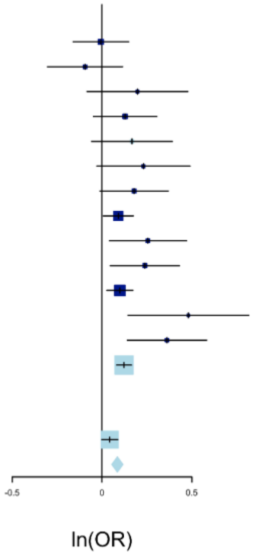

Heterogeneity discovery I^2 = *37.75%, p-value = 0.08*
Heterogeneity discovery + replication I^2 = *84.75%, p-value = 0.01*

CHR1:114377568
rs2476601: A/G

Cohort	N cases	ln(OR)	[95% CI]	p-value
**Discovery**				
BioVU	230	0.11	[-0.23; 0.45]	5.3e-01
UK Biobank	288	0.22	[-0.07; 0.51]	1.3e-01
DeCODE Genetics	133	0.29	[-0.07; 0.65]	1.2e-01
Seldin et al. 2015	89	0.55	[0.06; 1.04]	2.7e-02
Gregersen et al. 2012 mid EU	136	0.50	[0.10; 0.89]	1.4e-02
Estonian Biobank	206	0.44	[0.09; 0.78]	1.3e-02
FinnGen	329	0.30	[0.10; 0.50]	3.1e-03
Charite LUMC	390	0.40	[0.15; 0.64]	1.9e-03
Gregersen et al. 2012 ENG	276	0.52	[0.26; 0.79]	1.2e-04
Gregersen et al. 2012 north EU	228	0.58	[0.29; 0.86]	7.8e-05
Million Veterans Program	1,334	0.36	[0.23; 0.49]	3.5e-08
Chia et al. 2022	1,873	0.40	[0.28; 0.52]	7.9e-11
Discovery	5,708	0.38	[0.31; 0.44]	2.8e-31
**Replication**				
23andMe Replication	3,989	0.27	[0.20; 0.34]	7.8e-14
Discovery & Replication	9,697	0.33	[0.28; 0.38]	3.4e-43

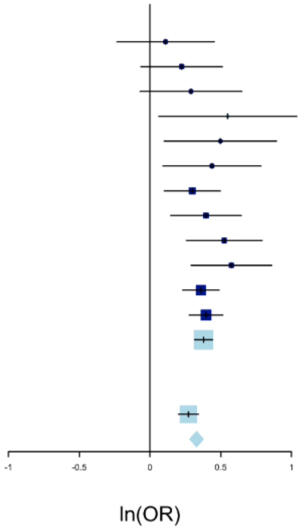

Heterogeneity discovery I^2 = *0%, p-value = 0.75*
Heterogeneity discovery + replication I^2 = *79.23%, p-value = 0.03*

CHR2:175616667
rs6433501: G/A

Cohort	N cases	ln(OR)	[95% CI]	p-value
**Discovery**				
Seldin et al. 2015	89	-0.07	[-0.58; 0.43]	7.8e-01
UK Biobank	288	0.04	[-0.24; 0.33]	7.6e-01
Gregersen et al. 2012 ENG	276	0.06	[-0.22; 0.34]	6.9e-01
Million Veterans Program	1,334	0.03	[-0.10; 0.16]	6.4e-01
DeCODE Genetics	133	-0.18	[-0.60; 0.24]	4.0e-01
Gregersen et al. 2012 north EU	228	0.16	[-0.16; 0.49]	3.2e-01
Estonian Biobank	206	0.25	[-0.10; 0.59]	1.7e-01
Topaloudi et al 2022	196	0.24	[-0.06; 0.54]	1.2e-01
FinnGen	329	0.23	[-0.04; 0.50]	9.2e-02
Gregersen et al. 2012 mid EU	136	0.36	[0.00; 0.71]	4.7e-02
Charite LUMC	390	0.32	[0.06; 0.57]	1.4e-02
Chia et al. 2022	1,873	0.22	[0.13; 0.32]	6.9e-06
Discovery	5,708	0.16	[0.10; 0.22]	3.5e-07
**Replication**				
23andMe Replication	3,989	0.14	[0.07; 0.20]	2.0e-05
Discovery & Replication	9,697	0.15	[0.10; 0.19]	2.7e-11

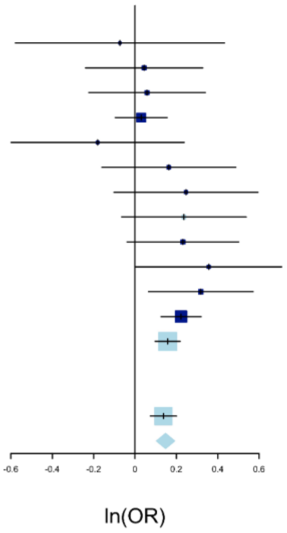

Heterogeneity discovery I^2 = *12.29%, p-value = 0.32*
Heterogeneity discovery + replication I^2 = *0%, p-value = 0.65*

CHR2:204729153
rs231770: T/C

Cohort	N cases	ln(OR)	[95% CI]	p-value
**Discovery**				
Topaloudi et al 2022	196	-0.00	[-0.24; 0.23]	9.7e-01
Gregersen et al. 2012 ENG	276	0.02	[-0.17; 0.21]	8.2e-01
Estonian Biobank	206	0.06	[-0.18; 0.29]	6.3e-01
Gregersen et al. 2012 mid EU	136	-0.11	[-0.40; 0.18]	4.7e-01
UK Biobank	288	0.08	[-0.11; 0.27]	4.2e-01
Gregersen et al. 2012 north EU	228	-0.10	[-0.31; 0.12]	3.9e-01
Seldin et al. 2015	89	-0.15	[-0.48; 0.18]	3.8e-01
BioVU	230	0.14	[-0.07; 0.35]	2.0e-01
DeCODE Genetics	133	0.18	[-0.08; 0.44]	1.8e-01
FinnGen	329	0.14	[-0.01; 0.29]	7.5e-02
Charite LUMC	390	0.24	[0.06; 0.41]	9.4e-03
Million Veterans Program	1,334	0.13	[0.04; 0.21]	2.8e-03
Chia et al. 2022	1,873	0.12	[0.04; 0.19]	2.5e-03
Discovery	5,708	0.10	[0.06; 0.14]	2.7e-06
**Replication**				
23andMe Replication	3,989	0.09	[0.05; 0.14]	4.7e-05
Discovery & Replication	9,697	0.10	[0.07; 0.13]	5.2e-10

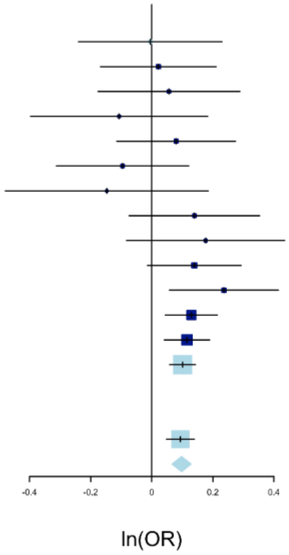

Heterogeneity discovery I^2 = *0%, p-value = 0.5*
Heterogeneity discovery + replication I^2 = *0%, p-value = 0.82*

CHR5:150447128
rs6861227: G/T

Cohort	N cases	ln(OR)	[95% CI]	p-value
Discovery				
Seldin et al. 2015	89	0.00	[-0.50; 0.50]	1.0e+00
Gregersen et al. 2012 mid EU	136	-0.06	[-0.42; 0.30]	7.4e-01
Topaloudi et al 2022	196	0.05	[-0.23; 0.33]	7.3e-01
Charite LUMC	390	-0.07	[-0.32; 0.18]	6.0e-01
DeCODE Genetics	133	0.13	[-0.26; 0.52]	5.1e-01
Million Veterans Program	1,334	0.05	[-0.07; 0.17]	4.3e-01
FinnGen	329	0.12	[-0.09; 0.34]	2.5e-01
Estonian Biobank	206	0.39	[0.05; 0.74]	2.6e-02
Chia et al. 2022	1,873	0.11	[0.02; 0.21]	2.3e-02
UK Biobank	288	0.34	[0.07; 0.60]	1.3e-02
BioVU	230	0.38	[0.10; 0.66]	7.4e-03
Gregersen et al. 2012 north EU	228	0.59	[0.30; 0.87]	5.1e-05
Gregersen et al. 2012 ENG	276	0.58	[0.34; 0.82]	1.7e-06
Discovery	5,708	0.15	[0.10; 0.21]	7.4e-08
Replication				
23andMe Replication	3,989	0.07	[0.01; 0.14]	2.1e-02
Discovery & Replication	9,697	0.12	[0.08; 0.16]	3.0e-08

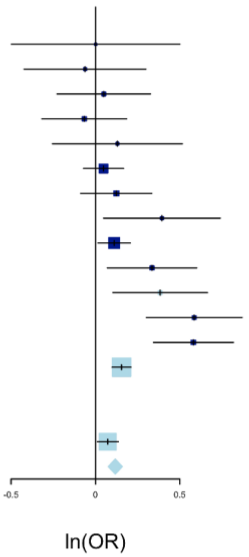

Heterogeneity discovery I^2 = *64.56%, p-value = 0*
Heterogeneity discovery + replication I^2 = *72.58%, p-value = 0.06*

CHR6:30095875
rs1264706: C/G

Cohort	N cases	ln(OR)	[95% CI]	p-value
Discovery				
BioVU	230	-0.00	[-0.33; 0.33]	9.9e-01
Seldin et al. 2015	89	-0.13	[-0.66; 0.39]	6.2e-01
Estonian Biobank	206	-0.13	[-0.61; 0.35]	6.1e-01
UK Biobank	288	-0.09	[-0.38; 0.21]	5.6e-01
Charite LUMC	390	0.19	[-0.10; 0.48]	2.0e-01
DeCODE Genetics	133	0.34	[-0.11; 0.79]	1.4e-01
FinnGen	329	0.43	[0.17; 0.69]	1.2e-03
Topaloudi et al 2022	196	0.86	[0.39; 1.32]	2.9e-04
Chia et al. 2022	1,873	0.30	[0.17; 0.42]	2.2e-06
Gregersen et al. 2012 mid EU	136	1.47	[1.08; 1.85]	1.2e-13
Gregersen et al. 2012 north EU	228	1.10	[0.81; 1.39]	7.1e-14
Gregersen et al. 2012 ENG	276	1.43	[1.19; 1.66]	2.4e-33
Discovery	5,708	0.48	[0.41; 0.56]	1.3e-34
Replication				
23andMe Replication	3,989	0.09	[0.00; 0.17]	3.8e-02
Discovery & Replication	9,697	0.30	[0.24; 0.35]	3.4e-25

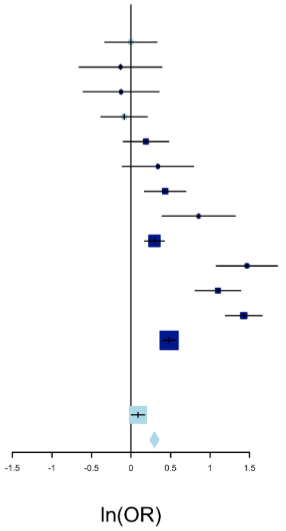

Heterogeneity discovery I^2 = *92.31%, p-value = 0*
Heterogeneity discovery + replication I^2 = *97.87%, p-value = 0*

CHR6:85513783
rs215918: A/G

Cohort	N cases	ln(OR)	[95% CI]	p-value
**Discovery**				
Estonian Biobank	206	0.05	[-0.18; 0.29]	6.5e-01
BioVU	230	0.06	[-0.16; 0.27]	6.0e-01
Charite LUMC	390	0.07	[-0.11; 0.25]	4.3e-01
Seldin et al. 2015	89	0.16	[-0.18; 0.50]	3.5e-01
Gregersen et al. 2012 mid EU	136	0.14	[-0.14; 0.41]	3.3e-01
Gregersen et al. 2012 ENG	276	0.10	[-0.09; 0.29]	3.1e-01
Gregersen et al. 2012 north EU	228	0.13	[-0.09; 0.35]	2.6e-01
DeCODE Genetics	133	0.17	[-0.11; 0.44]	2.3e-01
FinnGen	329	0.10	[-0.06; 0.25]	2.2e-01
Chia et al. 2022	1,873	0.08	[0.00; 0.15]	3.7e-02
UK Biobank	288	0.25	[0.05; 0.45]	1.2e-02
Topaloudi et al 2022	196	0.29	[0.07; 0.52]	1.2e-02
Million Veterans Program	1,334	0.15	[0.07; 0.24]	4.8e-04
Discovery	5,708	0.12	[0.08; 0.16]	3.4e-08
**Replication**				
23andMe Replication	3,989	0.06	[0.02; 0.11]	6.6e-03
Discovery & Replication	9,697	0.09	[0.06; 0.12]	3.8e-09

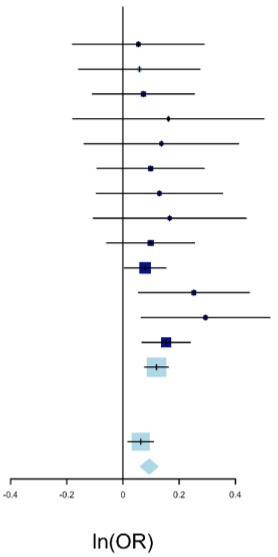

Heterogeneity discovery I^2 = *0%, p-value = 0.91*
Heterogeneity discovery + replication I^2 = *68.35%, p-value = 0.08*

CHR6:167437988
rs2301436: T/C

Cohort	N cases	ln(OR)	[95% CI]	p-value
**Discovery**				
UK Biobank	288	0.00	[-0.19; 0.20]	9.7e-01
Topaloudi et al 2022	196	-0.02	[-0.23; 0.20]	8.9e-01
Seldin et al. 2015	89	-0.07	[-0.40; 0.27]	6.9e-01
Gregersen et al. 2012 mid EU	136	0.07	[-0.19; 0.33]	5.9e-01
DeCODE Genetics	133	-0.08	[-0.33; 0.18]	5.7e-01
Estonian Biobank	206	0.14	[-0.09; 0.37]	2.2e-01
BioVU	230	0.20	[-0.01; 0.41]	6.7e-02
Chia et al. 2022	1,873	0.08	[0.00; 0.15]	4.0e-02
FinnGen	329	0.17	[0.02; 0.32]	3.1e-02
Gregersen et al. 2012 ENG	276	0.24	[0.06; 0.42]	1.1e-02
Million Veterans Program	1,334	0.11	[0.03; 0.20]	7.5e-03
Charite LUMC	390	0.24	[0.07; 0.42]	6.8e-03
Gregersen et al. 2012 north EU	228	0.32	[0.10; 0.54]	4.3e-03
Discovery	5,708	0.11	[0.07; 0.15]	1.1e-07
**Replication**				
23andMe Replication	3,989	0.06	[0.01; 0.10]	1.3e-02
Discovery & Replication	9,697	0.09	[0.06; 0.12]	2.3e-08

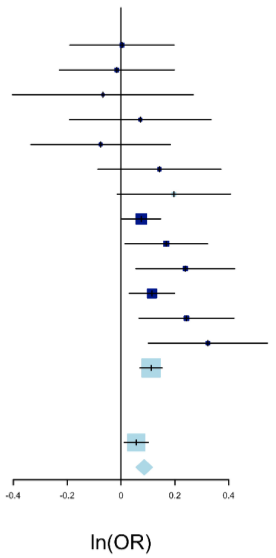

Heterogeneity discovery I^2 = *15.64%, p-value = 0.28*
Heterogeneity discovery + replication I^2 = *68.88%, p-value = 0.07*

CHR11:95311422
rs4409785: T/C

Cohort	N cases	ln(OR)	[95% CI]	p-value
Discovery				
DeCODE Genetics	133	-0.02	[-0.37; 0.33]	9.2e-01
Gregersen et al. 2012 north EU	228	-0.07	[-0.37; 0.22]	6.2e-01
Seldin et al. 2015	89	0.13	[-0.30; 0.56]	5.6e-01
UK Biobank	288	-0.08	[-0.33; 0.18]	5.6e-01
BioVU	230	0.15	[-0.12; 0.43]	2.8e-01
Estonian Biobank	206	0.18	[-0.13; 0.49]	2.5e-01
Topaloudi et al 2022	196	0.18	[-0.09; 0.44]	1.9e-01
Gregersen et al. 2012 mid EU	136	0.29	[-0.10; 0.67]	1.4e-01
Gregersen et al. 2012 ENG	276	0.21	[-0.05; 0.46]	1.1e-01
FinnGen	329	0.16	[-0.03; 0.36]	1.0e-01
Charite LUMC	390	0.21	[-0.02; 0.45]	7.5e-02
Million Veterans Program	1,334	0.15	[0.05; 0.26]	4.1e-03
Chia et al. 2022	1,873	0.25	[0.17; 0.34]	1.5e-08
Discovery	5,708	0.18	[0.13; 0.23]	2.7e-11
Replication				
23andMe Replication	3,989	0.08	[0.02; 0.14]	6.9e-03
Discovery & Replication	9,697	0.13	[0.09; 0.17]	1.5e-11

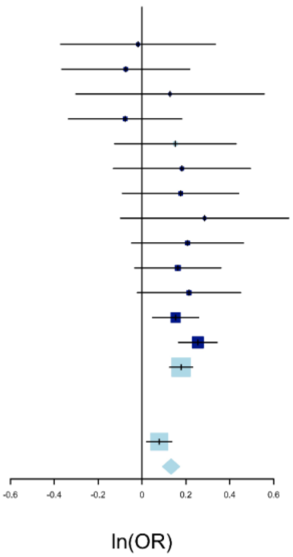

Heterogeneity discovery I^2 = *0%, p-value = 0.57*
Heterogeneity discovery + replication I^2 = *84.09%, p-value = 0.01*

CHR12:111904371
rs4766578: T/A

Cohort	N cases	ln(OR)	[95% CI]	p-value
Discovery				
DeCODE Genetics	133	0.05	[-0.21; 0.32]	6.9e-01
Estonian Biobank	206	-0.06	[-0.29; 0.17]	6.1e-01
UK Biobank	288	0.05	[-0.14; 0.25]	5.9e-01
Gregersen et al. 2012 north EU	228	0.10	[-0.12; 0.32]	3.8e-01
FinnGen	329	0.09	[-0.06; 0.25]	2.4e-01
Seldin et al. 2015	89	0.23	[-0.09; 0.55]	1.6e-01
Million Veterans Program	1,334	0.07	[-0.02; 0.15]	1.1e-01
Gregersen et al. 2012 mid EU	136	0.22	[-0.05; 0.49]	1.0e-01
Gregersen et al. 2012 ENG	276	0.18	[-0.02; 0.37]	8.0e-02
Topaloudi et al 2022	196	0.23	[0.01; 0.45]	3.8e-02
Charite LUMC	390	0.27	[0.09; 0.45]	3.3e-03
Chia et al. 2022	1,873	0.13	[0.06; 0.20]	3.5e-04
Discovery	5,708	0.12	[0.07; 0.16]	6.7e-08
Replication				
23andMe Replication	3,989	0.08	[0.04; 0.12]	4.2e-04
Discovery & Replication	9,697	0.10	[0.07; 0.13]	2.3e-10

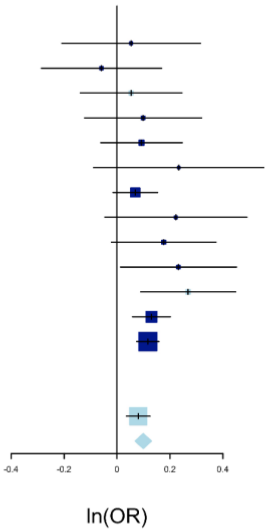

Heterogeneity discovery I^2 = *0%, p-value = 0.65*
Heterogeneity discovery + replication I^2 = *24.56%, p-value = 0.25*

CHR17:37912377
rs12946510: T/C

Cohort	N cases	ln(OR)	[95% CI]	p-value
**Discovery**				
BioVU	230	0.03	[-0.18; 0.25]	7.5e-01
Seldin et al. 2015	89	0.07	[-0.27; 0.41]	7.0e-01
UK Biobank	288	0.07	[-0.12; 0.26]	4.6e-01
Estonian Biobank	206	0.09	[-0.13; 0.32]	4.3e-01
Charite LUMC	390	0.09	[-0.09; 0.27]	3.1e-01
FinnGen	329	0.10	[-0.05; 0.26]	1.9e-01
Gregersen et al. 2012 mid EU	136	0.21	[-0.07; 0.48]	1.4e-01
Gregersen et al. 2012 north EU	228	0.18	[-0.04; 0.39]	1.1e-01
Topaloudi et al 2022	196	0.19	[-0.03; 0.40]	9.6e-02
Million Veterans Program	1,334	0.08	[-0.00; 0.17]	5.0e-02
Gregersen et al. 2012 ENG	276	0.24	[0.05; 0.43]	1.2e-02
DeCODE Genetics	133	0.37	[0.11; 0.63]	4.7e-03
Chia et al. 2022	1,873	0.17	[0.10; 0.24]	2.6e-06
Discovery	5,708	0.14	[0.10; 0.18]	7.6e-11
**Replication**				
23andMe Replication	3,989	0.08	[0.03; 0.12]	4.8e-04
Discovery & Replication	9,697	0.11	[0.08; 0.14]	8.3e-13

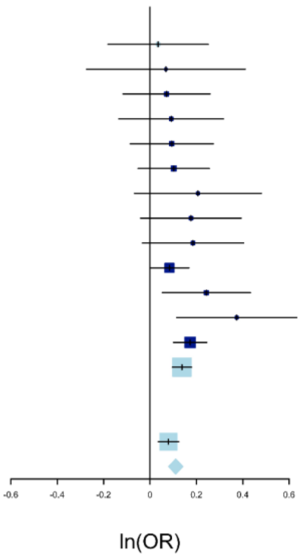

Heterogeneity discovery I^2 = 0%, p-value = 0.73
Heterogeneity discovery + replication I^2 = 71.17%, p-value = 0.06

CHR18:60005046
rs7239261: A/C

Cohort	N cases	ln(OR)	[95% CI]	p-value
**Discovery**				
Gregersen et al. 2012 mid EU	136	0.01	[-0.27; 0.30]	9.3e-01
FinnGen	329	0.03	[-0.13; 0.19]	7.3e-01
Gregersen et al. 2012 ENG	276	-0.04	[-0.23; 0.16]	6.9e-01
Gregersen et al. 2012 north EU	228	-0.09	[-0.30; 0.13]	4.3e-01
Charite LUMC	390	0.15	[-0.02; 0.33]	9.3e-02
Estonian Biobank	206	0.21	[-0.03; 0.45]	8.1e-02
BioVU	230	0.20	[-0.01; 0.40]	6.0e-02
DeCODE Genetics	133	0.36	[0.10; 0.61]	6.2e-03
Seldin et al. 2015	89	0.47	[0.14; 0.79]	4.5e-03
UK Biobank	288	0.28	[0.09; 0.47]	3.7e-03
Topaloudi et al 2022	196	0.35	[0.13; 0.57]	2.1e-03
Million Veterans Program	1,334	0.19	[0.10; 0.27]	1.2e-05
Chia et al. 2022	1,873	0.26	[0.19; 0.33]	1.8e-12
Discovery	5,708	0.19	[0.15; 0.24]	4.0e-20
**Replication**				
23andMe Replication	3,989	0.08	[0.03; 0.12]	8.9e-04
Discovery & Replication	9,697	0.14	[0.11; 0.17]	2.5e-19

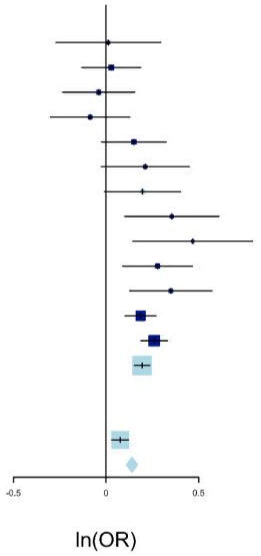

Heterogeneity discovery I^2 = 54.05%, p-value = 0.01
Heterogeneity discovery + replication I^2 = 93.03%, p-value = 0

Forest plots displaying the effect sizes ln(OR) and confidence intervals (95%; error bars) across all cohorts in the discovery sample, the replication, and the overall meta-analysis of discovery and replication GWASs.

Supplementary Figure 4. Forest plots of index SNPs across AChR and mixed MG samples

CHR1:114217705
rs7522138: A/G

Cohort	N cases	ln(OR)	[95% CI]	p-value
AChR antibody only	2,798	0.14	[0.08; 0.20]	4.0e-06
All antibody profiles	2,910	0.11	[0.05; 0.16]	3.1e-04

Heterogeneity: I^2 = 0%, τ^2 = 0, p = 4.15e-01

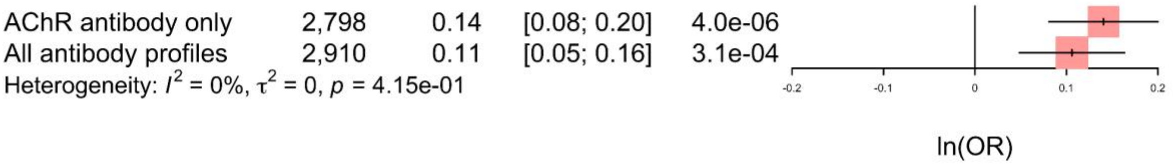

CHR1:114377568
rs2476601: A/G

Cohort	N cases	ln(OR)	[95% CI]	p-value
AChR antibody only	2,798	0.45	[0.35; 0.54]	1.7e-19
All antibody profiles	2,910	0.33	[0.24; 0.41]	4.3e-14

Heterogeneity: I^2 = 70%, τ^2 = 0.0049, p = 6.96e-02

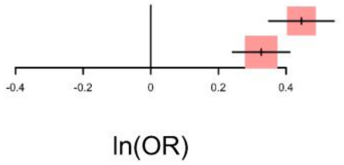

CHR2:175616667
rs6433501: G/A

Cohort	N cases	ln(OR)	[95% CI]	p-value
AChR antibody only	2,798	0.21	[0.12; 0.29]	7.0e-07
All antibody profiles	2,910	0.10	[0.01; 0.19]	3.7e-02

Heterogeneity: I^2 = 67%, τ^2 = 0.0039, p = 8.22e-02

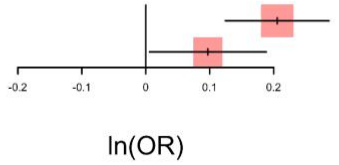

CHR2:204734487
rs231779: T/C

Cohort	N cases	ln(OR)	[95% CI]	p-value
AChR antibody only	2,798	0.06	[0.00; 0.13]	4.1e-02
All antibody profiles	2,910	0.14	[0.08; 0.20]	2.1e-06

Heterogeneity: I^2 = 68%, τ^2 = 0.0020, p = 7.81e-02

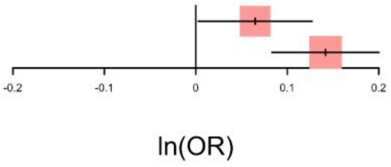

CHR5:150447128
rs6861227: G/T

Cohort	N cases	ln(OR)	[95% CI]	p-value
AChR antibody only	2,798	0.18	[0.10; 0.26]	4.9e-06
All antibody profiles	2,910	0.13	[0.04; 0.21]	2.6e-03

Heterogeneity: I^2 = 0%, τ^2 = 0, p = 3.19e-01

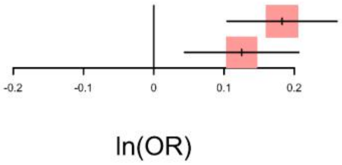

CHR6:30095875
rs1264706: C/G

Cohort	N cases	ln(OR)	[95% CI]	p-value
AChR antibody only	2,798	0.65	[0.56; 0.75]	2.3e-41
All antibody profiles	2,910	0.15	[0.02; 0.28]	2.4e-02

Heterogeneity: I^2 = 97%, τ^2 = 0.1206, p = 2.03e-09

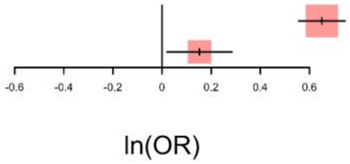

CHR6:85513783
rs215918: A/G

Cohort	N cases	ln(OR)	[95% CI]	p-value
AChR antibody only	2,798	0.10	[0.04; 0.17]	6.9e-04
All antibody profiles	2,910	0.13	[0.07; 0.19]	1.1e-05

Heterogeneity: I^2 = 0%, τ^2 = 0, p = 5.16e-01

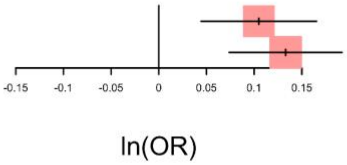

CHR6:167437988
rs2301436: T/C

Cohort	N cases	ln(OR)	[95% CI]	p-value
AChR antibody only	2,798	0.10	[0.04; 0.16]	1.1e-03
All antibody profiles	2,910	0.12	[0.07; 0.18]	2.3e-05

Heterogeneity: I^2 = 0%, τ^2 = 0, p = 5.29e-01

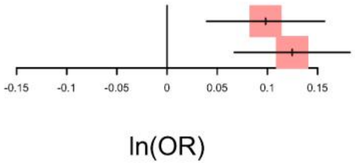

CHR11:95311422
rs4409785: T/C

Cohort	N cases	ln(OR)	[95% CI]	p-value
AChR antibody only	2,798	0.22	[0.15; 0.29]	5.4e-09
All antibody profiles	2,910	0.14	[0.06; 0.21]	3.4e-04

Heterogeneity: I^2 = 60%, τ^2 = 0.0022, p = 1.12e-01

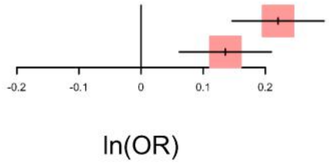

CHR12:111904371
rs4766578: T/A

Cohort	N cases	ln(OR)	[95% CI]	p-value
AChR antibody only	2,798	0.15	[0.09; 0.21]	1.0e-06
All antibody profiles	2,910	0.08	[0.02; 0.14]	6.4e-03

Heterogeneity: I^2 = 54%, τ^2 = 0.0011, p = 1.41e-01

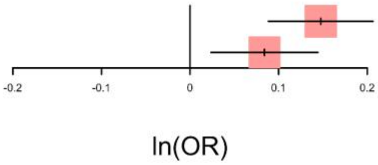

CHR17:37912377
rs12946510: T/C

Cohort	N cases	ln(OR)	[95% CI]	p-value
AChR antibody only	2,798	0.18	[0.12; 0.24]	3.2e-09
All antibody profiles	2,910	0.10	[0.04; 0.16]	9.1e-04

Heterogeneity: I^2 = 73%, τ^2 = 0.0024, p = 5.38e-02

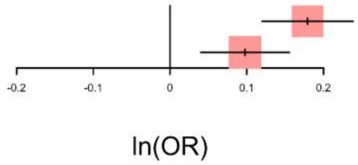

CHR18:60005046
rs7239261: A/C

Cohort	N cases	ln(OR)	[95% CI]	p-value
AChR antibody only	2,798	1.23	[0.88; 1.58]	7.9e-12
All antibody profiles	2,910	1.20	[0.82; 1.58]	7.0e-10

Heterogeneity: I^2 = 0%, τ^2 = 0, p = 9.04e-01

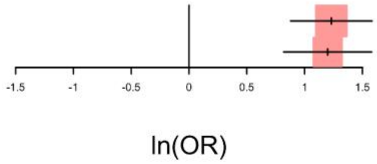

Forest plots displaying the effect sizes ln(OR) and confidence intervals (95%; error bars) across cohorts filtered for acetylcholine receptor (AChR) antibody-positive cases and cohorts not filtered for specific antibody configurations.

Supplementary Figure 5. Region plots of EOMG discovery GWAS

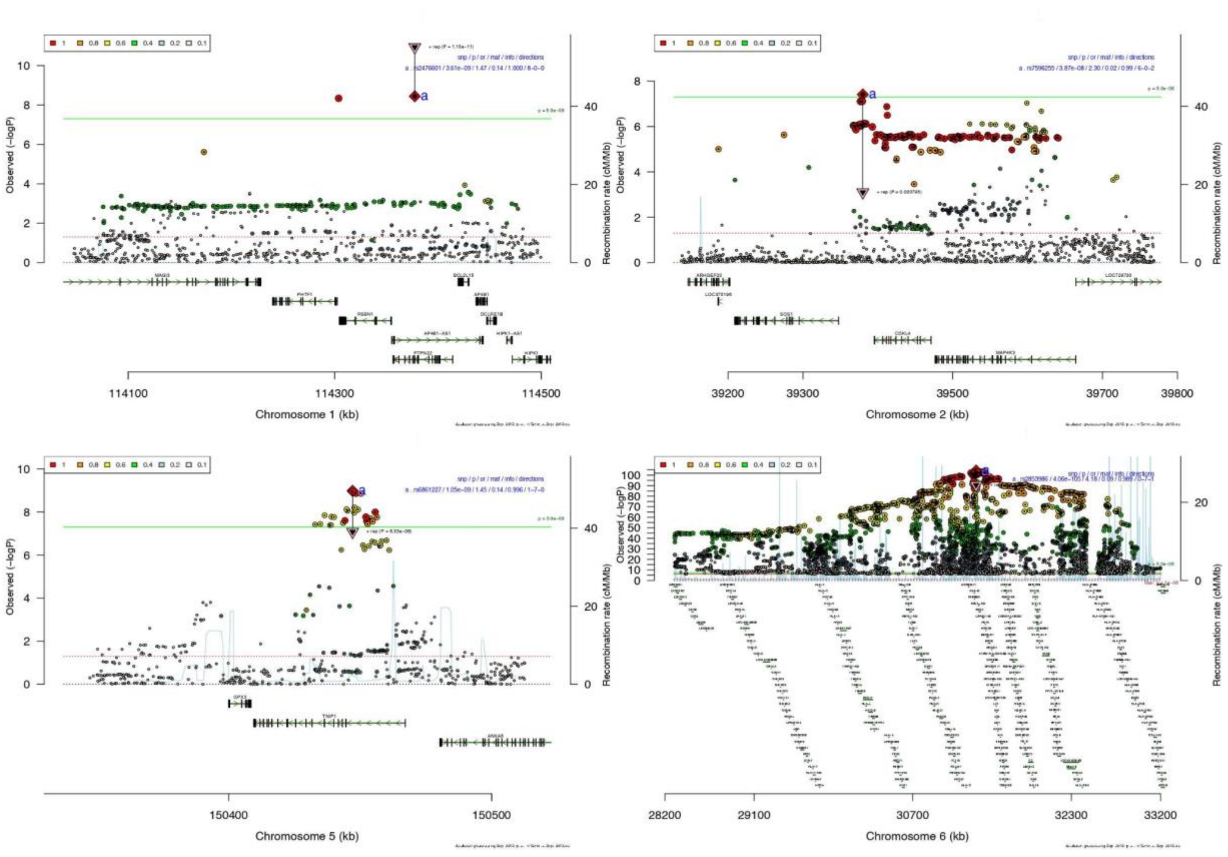

Each point in the plot represents a SNP that is colored according to the level of linkage disequilibrium with the index SNP. The left x-axis depicts the genomic position and the y-axis indicates the observed -log10 p-value. The right y-axis shows the Recombination rate (cM/Mb). The green line indicates the genome-wide significance threshold of *p*<5e-8. Triangles indicate the index SNPs p-value in the discovery and replication meta-analysis.

Supplementary Figure 6. Forest plots of index SNPs for EOMG

CHR1:114377568
rs2476601: A/G

Cohort	N cases	ln(OR)	[95% CI]	p-value
Discovery				
Estonian Biobank	101	0.01	[-0.50; 0.51]	9.7e-01
Charite LUMC	176	0.15	[-0.20; 0.50]	4.1e-01
Renton et al. 2015	264	0.24	[-0.24; 0.72]	3.2e-01
FinnGen	136	0.24	[-0.07; 0.55]	1.4e-01
Million Veterans Program	74	0.65	[-0.08; 1.39]	8.0e-02
Gregersen et al. 2012 mid EU	136	0.50	[0.10; 0.89]	1.4e-02
Gregersen et al. 2012 ENG	276	0.52	[0.26; 0.79]	1.2e-04
Gregersen et al. 2012 north EU	228	0.58	[0.29; 0.86]	7.8e-05
Discovery	1,391	0.39	[0.26; 0.51]	3.6e-09
Replication				
23andMe Replication	871	0.27	[0.12; 0.42]	5.5e-04
Discovery & Replication	2,262	0.33	[0.24; 0.43]	1.2e-11

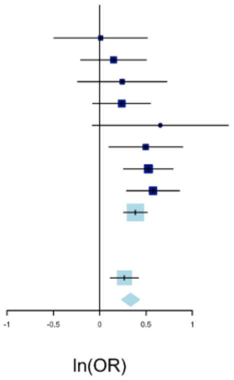

Heterogeneity discovery I^2= 7.25%, *p-value* = 0.37
Heterogeneity discovery + replication I^2 = 28.07%, *p-value* = 0.24

CHR6:31338844
rs2853986: C/T

Cohort	N cases	ln(OR)	[95% CI]	p-value
Discovery				
Estonian Biobank	101	0.52	[-0.15; 1.19]	1.3e-01
Charite LUMC	176	0.78	[0.43; 1.12]	9.0e-06
Renton et al. 2015	264	1.32	[0.88; 1.75]	3.3e-09
FinnGen	136	1.10	[0.80; 1.40]	4.6e-13
Gregersen et al. 2012 mid EU	136	1.98	[1.58; 2.37]	1.4e-22
Gregersen et al. 2012 north EU	228	1.48	[1.19; 1.76]	1.6e-24
Gregersen et al. 2012 ENG	276	1.98	[1.72; 2.25]	1.3e-48
Discovery	1,391	1.43	[1.30; 1.56]	4.1e-105
Replication				
23andMe Replication	871	0.45	[0.32; 0.58]	2.6e-11
Discovery & Replication	2,262	0.93	[0.84; 1.02]	2.7e-91

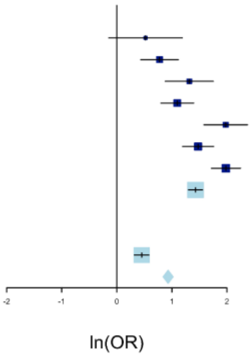

Heterogeneity discovery I^2= 86.05%, *p-value* = 0
Heterogeneity discovery + replication I^2 = 99.07%, *p-value* = 0

Forest plots displaying the effect sizes ln(OR) and confidence intervals (95%; error bars) across all cohorts in the discovery sample, the replication, and the overall meta-analysis of discovery and replication GWASs.

Supplementary Figure 7. Region plots of LOMG discovery GWAS

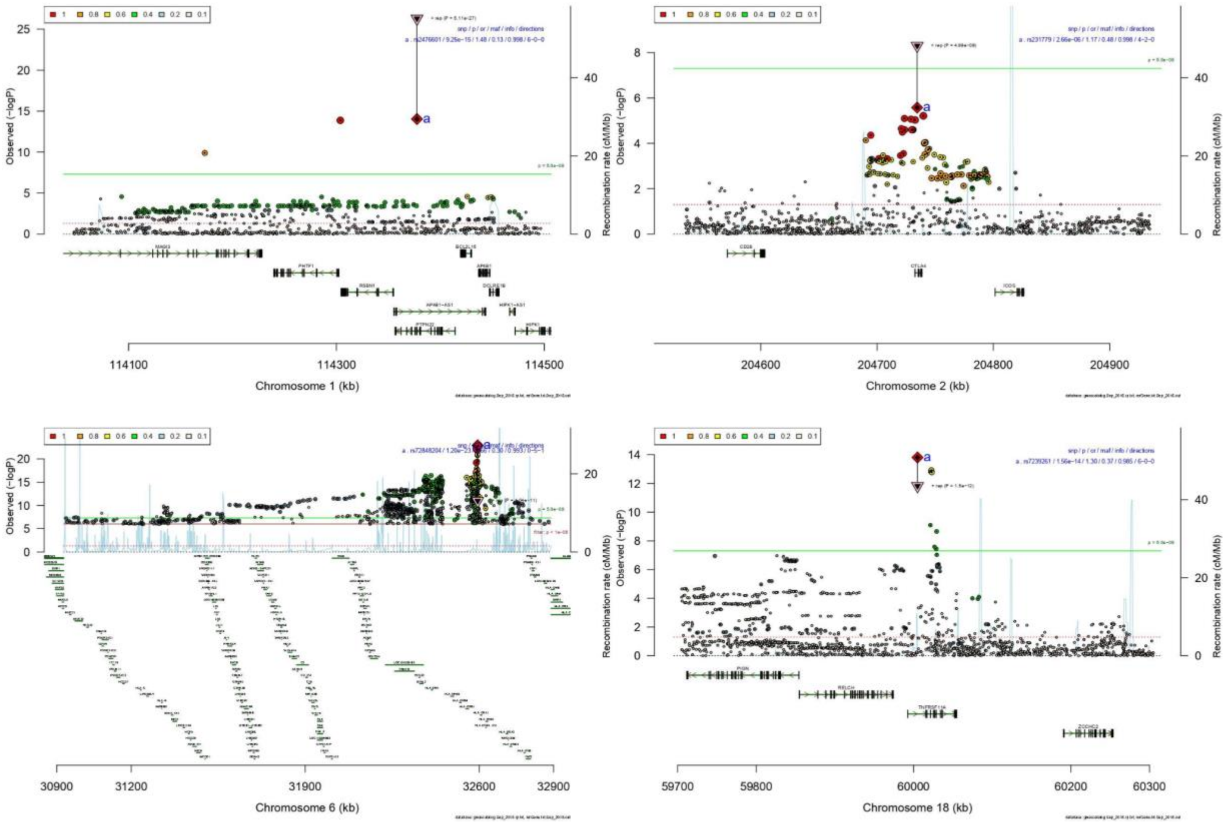

Each point in the plot represents a SNP that is colored according to the level of linkage disequilibrium with the index SNP. The left x-axis depicts the genomic position and the y-axis indicates the observed -log10 p-value. The right y-axis shows the Recombination rate (cM/Mb). The green line indicates the genome-wide significance threshold of *p*<5e-8. Triangles indicate the index SNPs p-value in the discovery and replication meta-analysis.

Supplementary Figure 8. Forest plots of index SNPs for LOMG

CHR1:114377568
rs2476601: A/G

Cohort	N cases	ln(OR)	[95% CI]	p-value
**Discovery**				
Estonian Biobank	105	0.52	[0.04; 0.99]	3.3e-02
Seldin et al. 2015	89	0.55	[0.06; 1.04]	2.7e-02
FinnGen	193	0.32	[0.07; 0.58]	1.4e-02
Charite & Leiden UMC	213	0.89	[0.22; 1.57]	9.5e-03
Renton et al. 2015	544	0.38	[0.16; 0.60]	7.1e-04
Million Veterans Program	1,260	0.37	[0.24; 0.50]	5.4e-08
Discovery	2,404	0.39	[0.29; 0.49]	9.3e-15
**Replication**				
23andMe Replication	3,989	0.27	[0.20; 0.34]	7.8e-14
Discovery & Replication	6,393	0.31	[0.25; 0.37]	5.1e-27

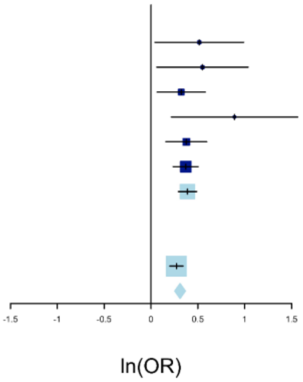

Heterogeneity discovery I^2 = *0%, p-value = 0.79*
Heterogeneity discovery + replication I^2 = *72.61%, p-value = 0.06*

CHR2:204729153
rs231770: T/C

Cohort	N cases	ln(OR)	[95% CI]	p-value
**Discovery**				
Estonian Biobank	105	-0.11	[-0.45; 0.23]	5.3e-01
Seldin et al. 2015	89	-0.15	[-0.48; 0.18]	3.8e-01
Charite & Leiden UMC	213	0.18	[-0.22; 0.59]	3.7e-01
FinnGen	193	0.17	[-0.03; 0.37]	1.0e-01
Million Veterans Program	1,260	0.13	[0.05; 0.22]	2.8e-03
Renton et al. 2015	544	0.28	[0.14; 0.42]	1.1e-04
Discovery	2,404	0.15	[0.08; 0.22]	8.6e-06
**Replication**				
23andMe Replication	3,989	0.09	[0.05; 0.14]	4.7e-05
Discovery & Replication	6,393	0.11	[0.07; 0.15]	4.2e-09

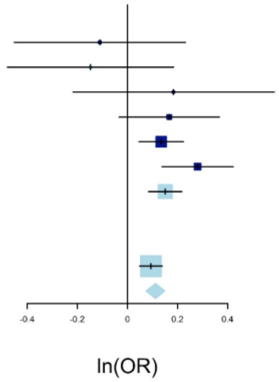

Heterogeneity discovery I^2 = *31.04%, p-value = 0.19*
Heterogeneity discovery + replication I^2 = *48.49%, p-value = 0.16*

CHR6:32594073
rs72848204: G/T

Cohort	N cases	ln(OR)	[95% CI]	p-value
**Discovery**				
Charite & Leiden UMC	213	0.36	[-0.11; 0.83]	1.3e-01
FinnGen	193	0.22	[-0.01; 0.45]	5.8e-02
Seldin et al. 2015	89	0.50	[-0.01; 1.00]	5.5e-02
Million Veterans Program	1,260	0.32	[0.22; 0.43]	4.7e-10
Renton et al. 2015	544	0.79	[0.61; 0.97]	4.2e-18
Discovery	2,404	0.41	[0.33; 0.49]	1.2e-23
**Replication**				
23andMe Replication	3,989	0.05	[0.00; 0.10]	4.7e-02
Discovery & Replication	6,393	0.14	[0.10; 0.18]	1.0e-11

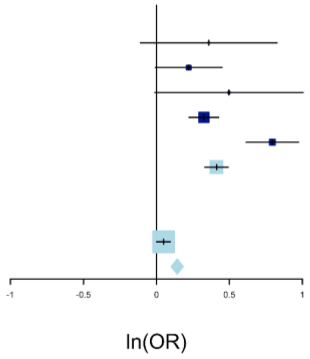

Heterogeneity discovery I^2 = *78.32%, p-value = 0*
Heterogeneity discovery + replication I^2 = *98.28%, p-value = 0*

CHR18:60005046
rs7239261: A/C

Cohort	N cases	ln(OR)	[95% CI]	p-value
**Discovery**				
Estonian Biobank	105	0.04	[-0.29; 0.38]	8.0e-01
Charite & Leiden UMC	213	0.31	[-0.11; 0.74]	1.4e-01
FinnGen	193	0.24	[0.03; 0.44]	2.2e-02
Seldin et al. 2015	89	0.47	[0.14; 0.79]	4.5e-03
Million Veterans Program	1,260	0.19	[0.11; 0.28]	1.4e-05
Renton et al. 2015	544	0.44	[0.30; 0.59]	1.6e-09
Discovery	2,404	0.26	[0.19; 0.33]	1.6e-14
**Replication**				
23andMe Replication	3,989	0.08	[0.03; 0.12]	8.9e-04
Discovery & Replication	6,393	0.13	[0.10; 0.17]	1.5e-12

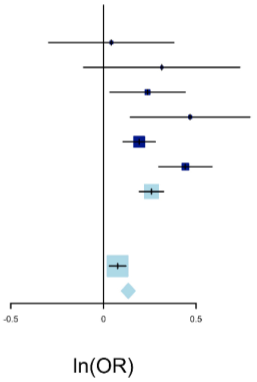

Heterogeneity discovery I^2 = *49.04%, p-value = 0.07*
Heterogeneity discovery + replication I^2 = *95.03%, p-value = 0*

Forest plots displaying the effect sizes ln(OR) and confidence intervals (95%; error bars) across all cohorts in the discovery sample, the replication, and the overall meta-analysis of discovery and replication GWASs.

Supplementary Figure 9. Region plots of imputed HLA types in the combined GWAS

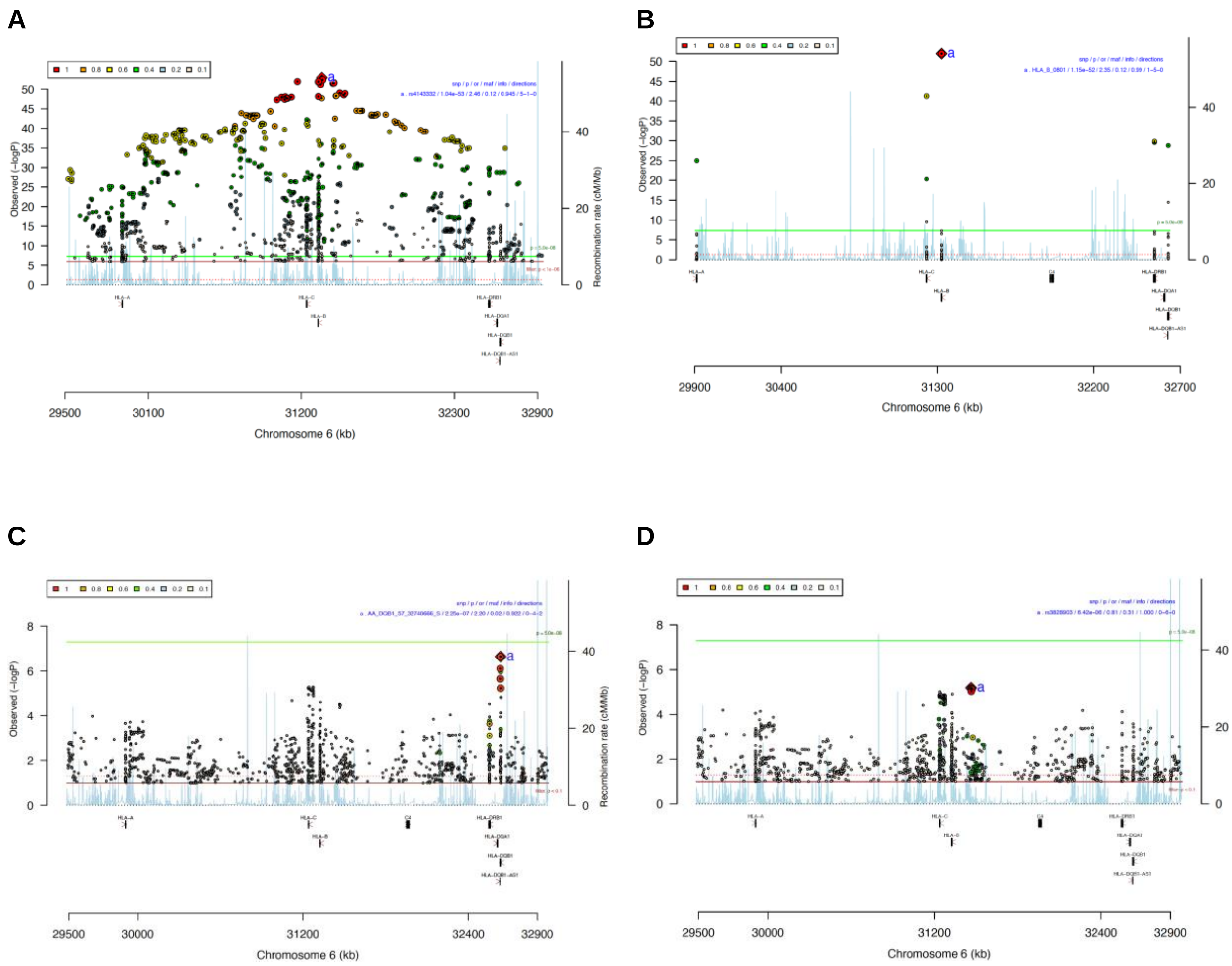

A Full MHC region after imputing HLA types. **B** Region plot of the MHC filtering only HLA encoding alleles. **C** Area plot of the MHC after conditioning on rs4143332 **D** Area plot of the MHC region after conditioning on rs4143332 and AA-DQB1_57_32740666_S. The left x-axis depicts the genomic position and the y-axis indicates the observed -log10 p-value. The right y-axis shows the Recombination rate (cM/Mb). The green line indicates the genome-wide significance threshold of p<5e-8.

Supplementary Figure 10. Region plots of imputed HLA types in the early-onset GWAS

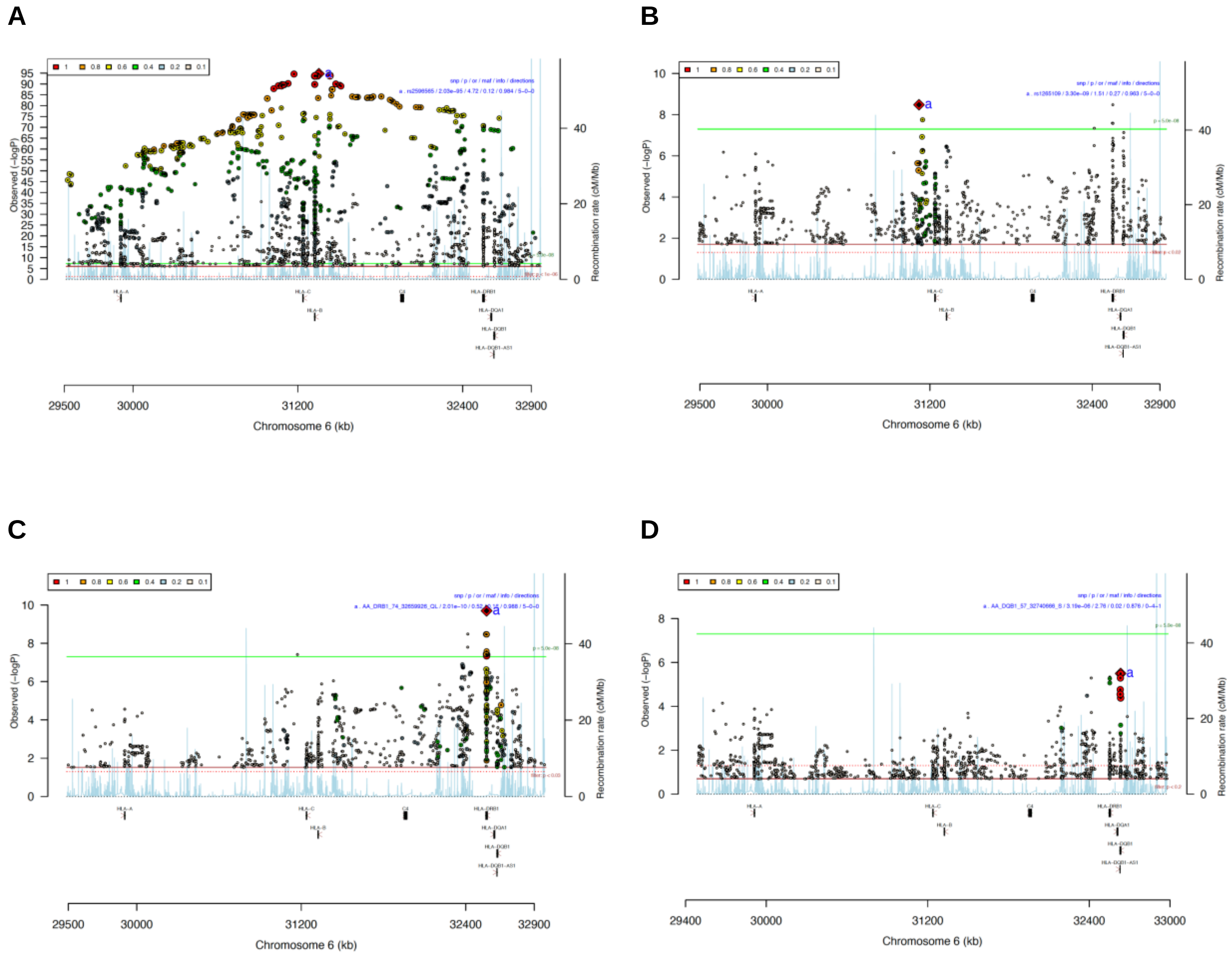

A Full MHC region after imputing HLA types. **B** Region plot of the MHC after conditioning on the top variant rs2596565 revealing two independent significant signals at HLA-B and HLA-DRB1 **C** Region plot of the MHC after conditioning on rs2596565 and rs1265109 showing a distinct significant signal of HLA-DRB1*07:01 **D** Region plot of the MHC region after conditioning on rs2596565, rs1265109, and AA_DRB1_74_32659926_QL showing now remaining significant signals. The left x-axis depicts the genomic position and the y-axis indicates the observed -log10 p-value. The right y-axis shows the Recombination rate (cM/Mb). The green line indicates the genome-wide significance threshold of p<5e-8.

Supplementary Figure 11. Region plots of imputed HLA types in the late-onset GWAS

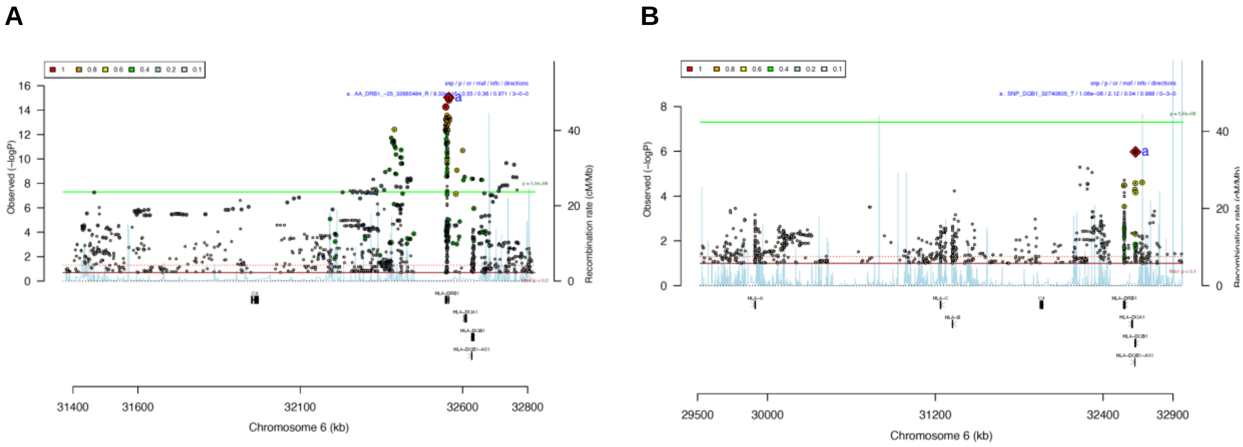

A Full MHC region after imputing HLA types showing a genome-wide significant signal at HLA-DRB1. **B** Region plot of the MHC after conditioning on the AA_DRB1_25_32665484_R showing now remaining distinct significant loci. The left x-axis depicts the genomic position and the y-axis indicates the observed -log10 p-value. The right y-axis shows the Recombination rate (cM/Mb). The green line indicates the genome-wide significance threshold of *p*<5e-8.

Supplementary Figure 12. Manhattan plot of TWAS results for skeletal muscle tissue

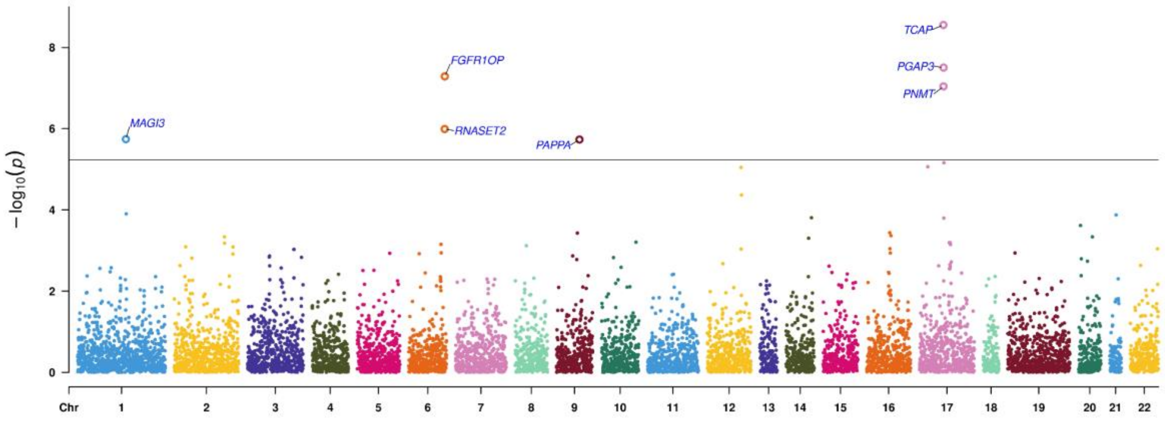

Gene-level Manhattan plot showing the results of the TWAS analysis for skeletal muscle tissue excluding the extended MHC region (25-35Mb). The x-axis shows the chromosome positions in ascending order. The y-axis displays the -log10 p-values of Genes names are plotted for genes below a Bonferroni corrected p-value threshold of < 5.81^{-6}.

Supplementary Figure 13. Manhattan plot of TWAS results for tibial nerve tissue

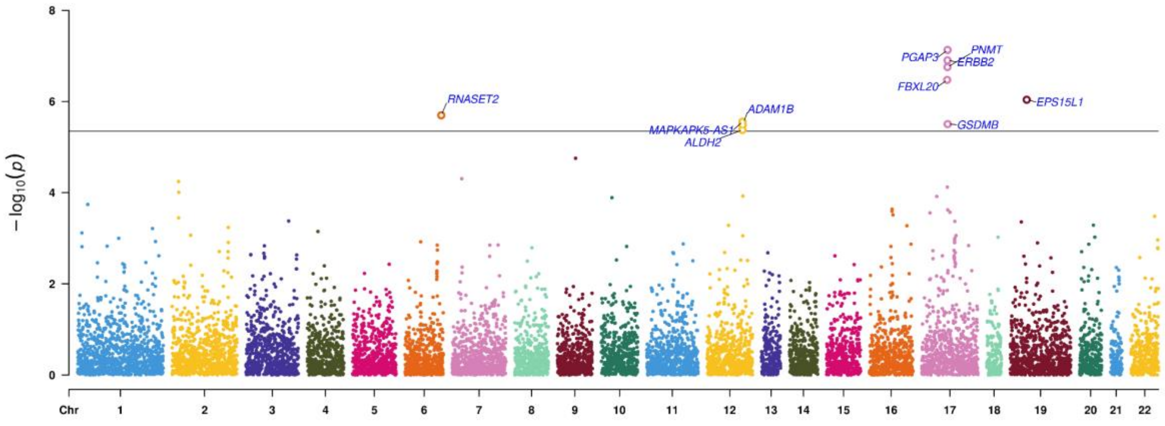

Gene-level Manhattan plot showing the results of the TWAS analysis for tibial nerve tissue excluding the extended MHC region (25-35Mb). The x-axis shows the chromosome positions in ascending order. The y-axis displays the -log10 p-values of Genes names are plotted for genes below a Bonferroni corrected p-value threshold of < 4.40^{-6}.

Supplementary Figure 14. Manhattan plot of TWAS results for whole blood tissue

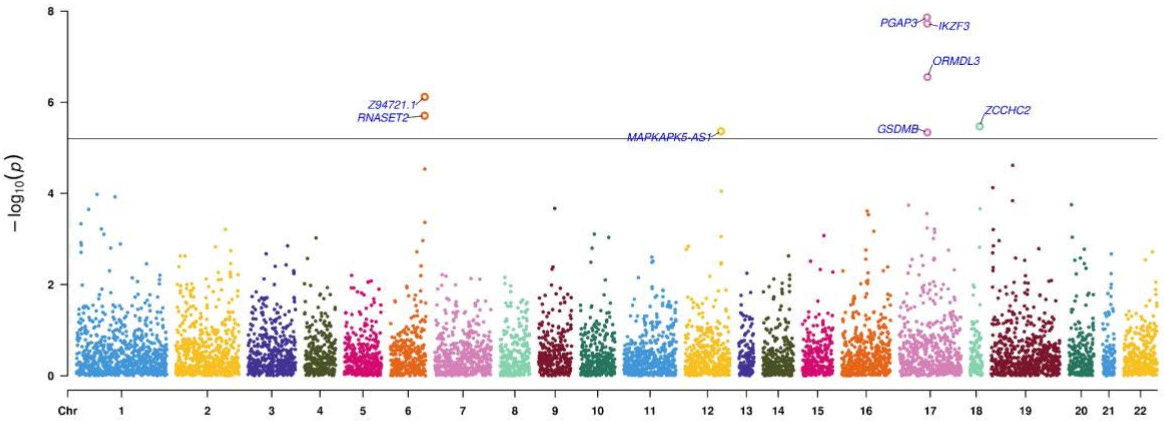

Gene-level Manhattan plot showing the results of the TWAS analysis for whole blood. The x-axis shows the chromosome positions in ascending order excluding the extended MHC region (25-35Mb). The y-axis displays the -log10 p-values of Genes names are plotted for genes below a Bonferroni corrected p-value threshold of $< 6.20e^{-6}$.

Supplementary Figure 15. Performance of MG PRS across onset phenotypes

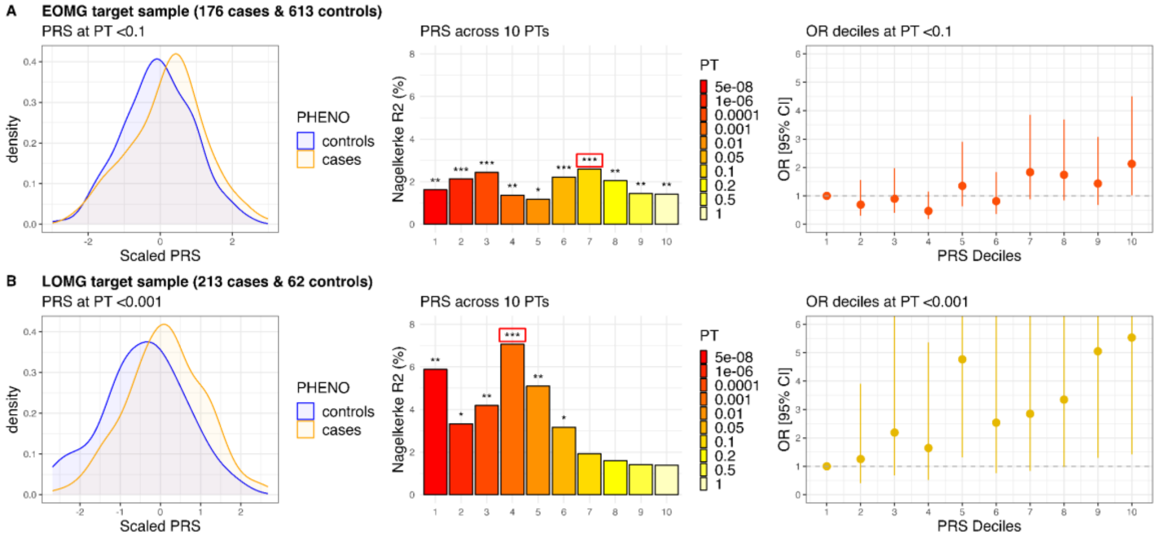

Results of polygenic risk scoring of **A:** early-onset MG, and **B:** late-onset MG target sample of CCM and LUMC cases and controls across all 10 PTs along with density and decile plots for the best performing PT. All samples were scored using the combined MG leave-one-out training dataset due to the larger sample size and power.

Supplementary Figure 16. Performance of MG PRS across antibody phenotypes

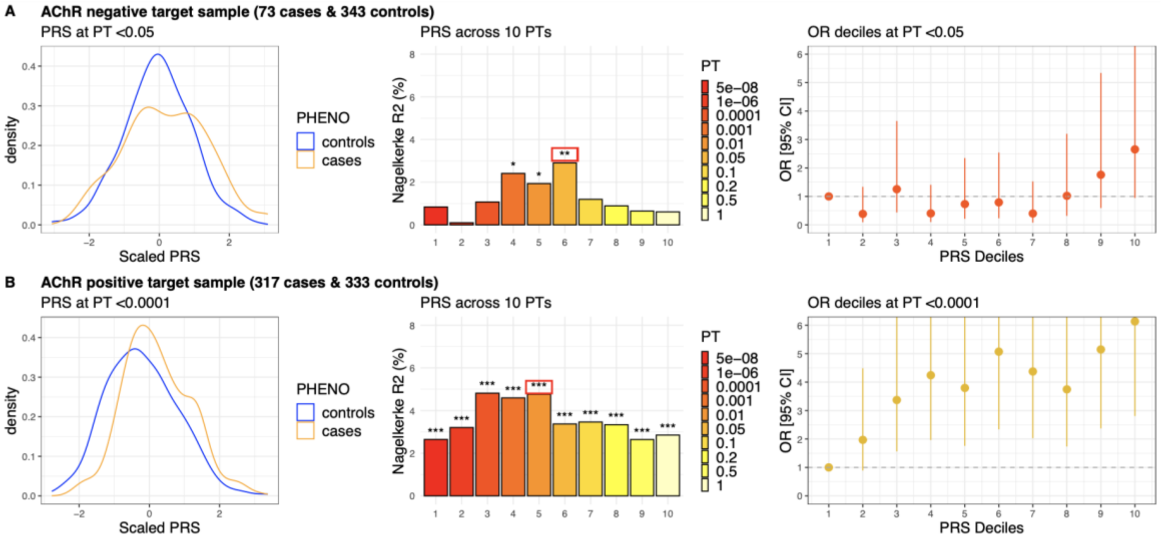

Results of polygenic risk scoring of **A:** AChR antibody negative, and **B:** AChR antibody positive target sample of CCM and LUMC cases and controls across all 10 PTs along with density and decile plots for the best performing PT. All samples were scored using the combined MG leave-one-out training dataset due to the larger sample size and power.

Supplementary Figure 17. Genetic correlation results of pre-selected traits

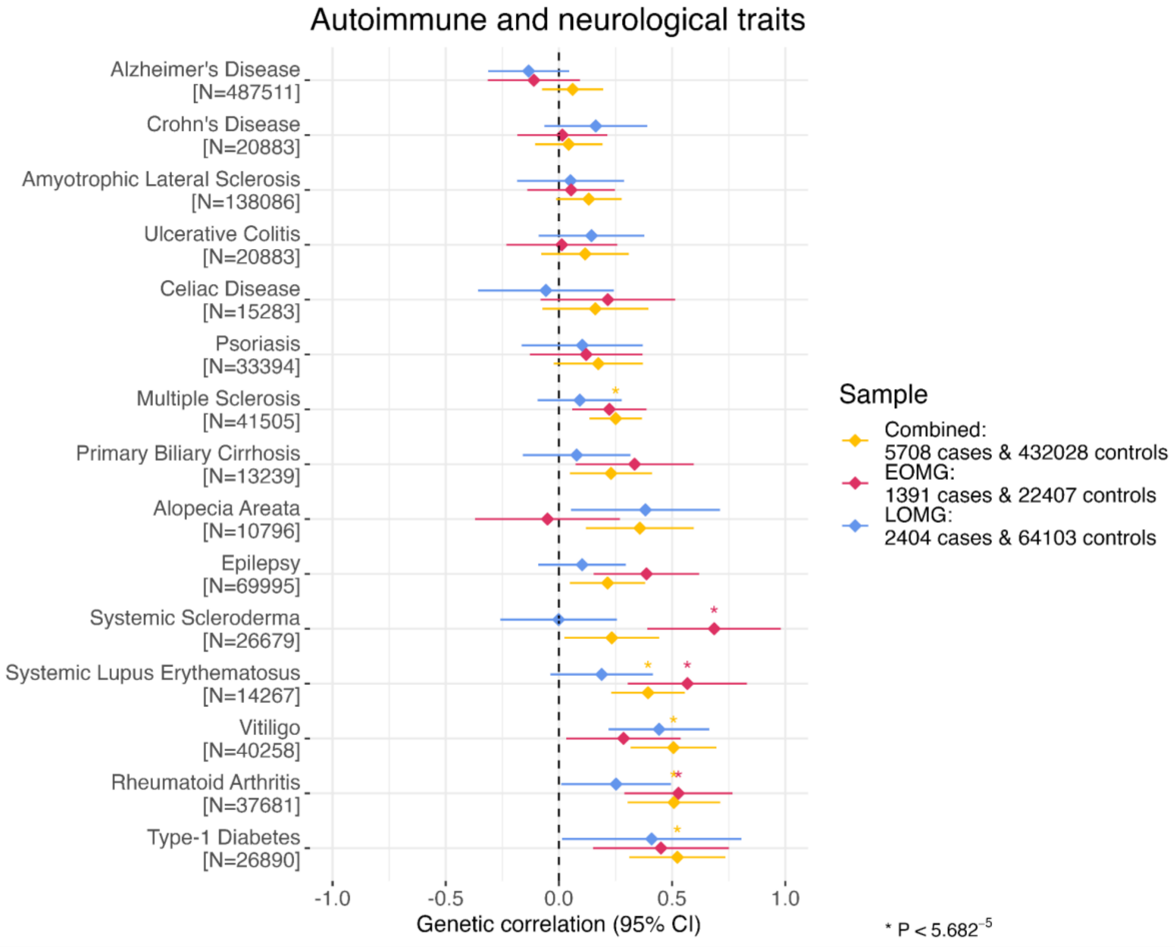

The forest plot gives an overview of the genetic correlation coefficient r_g and the 95% confidence interval (error bars) of MG, EOMG, and LOMG with other neurological and autoimmune traits calculated through LDSC. Asterisks indicate statistical significance below the Bonferroni-corrected p-value, taking into account all 880 tests conducted across pre-selected and FinnGen release 8 traits.

Supplementary Figure 18. Variant lookup in UK Biobank, GWAS Catalog and FinnGen

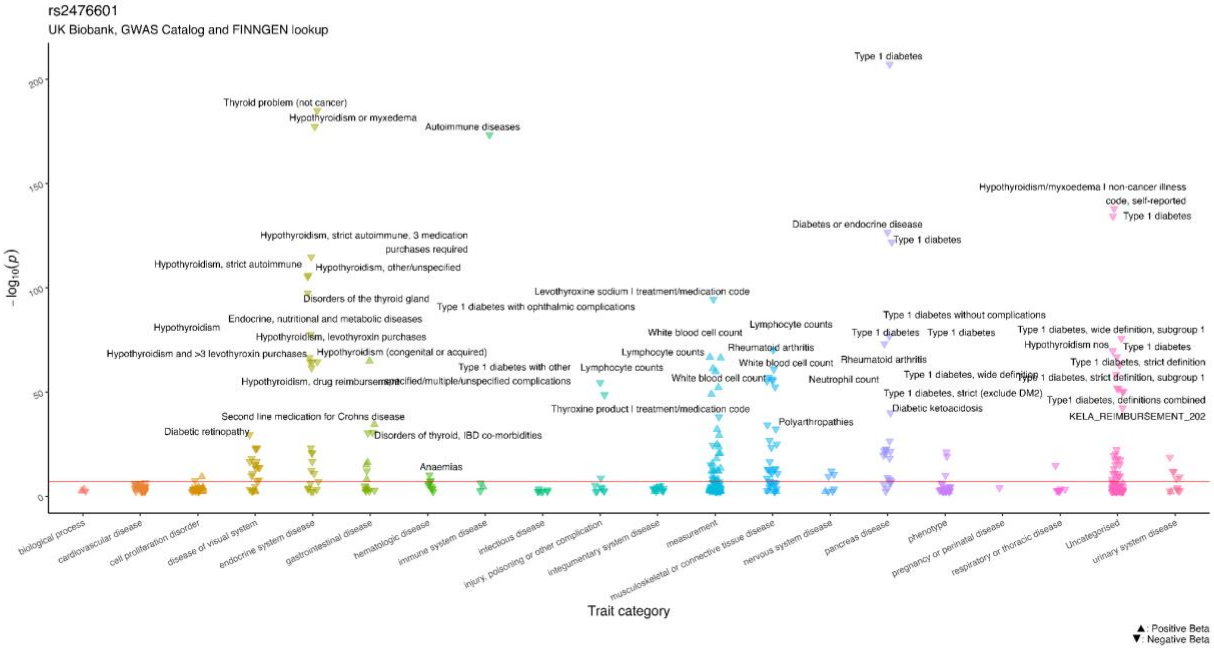

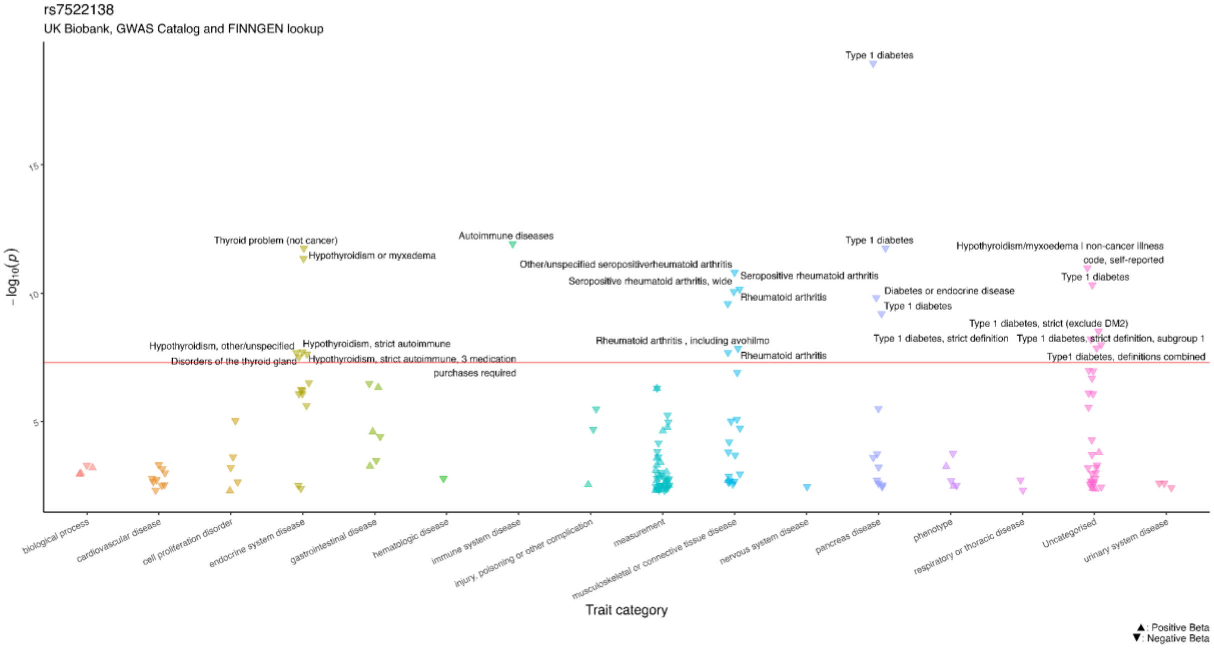

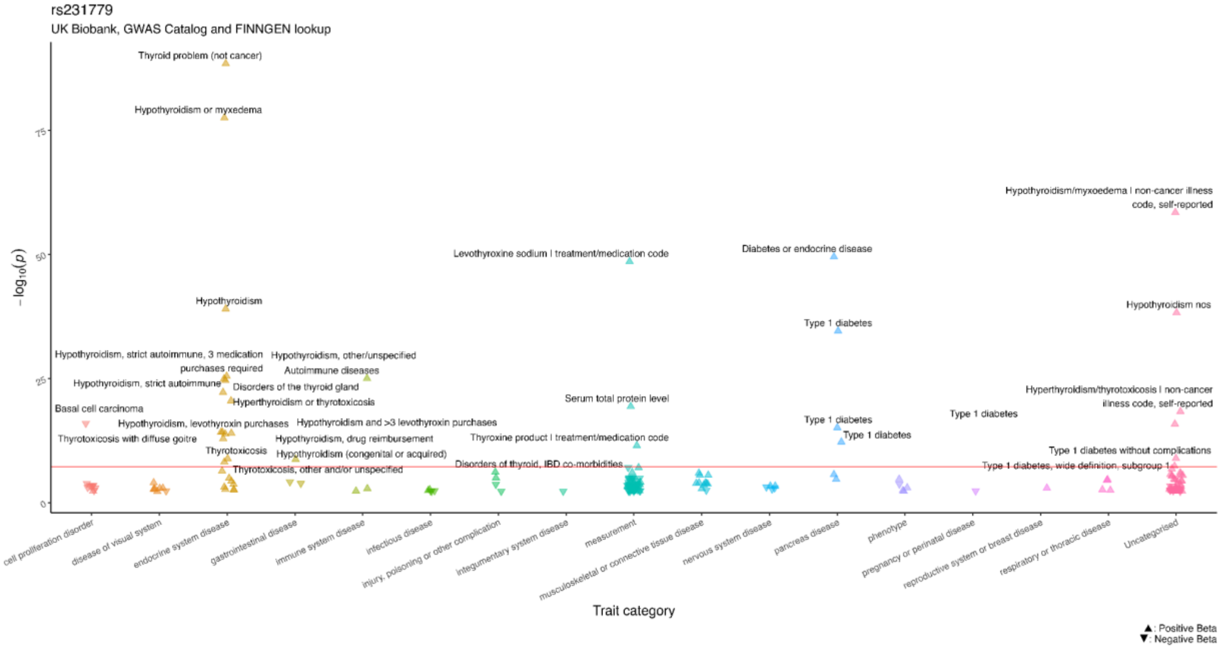
rs231779
UK Biobank, GWAS Catalog and FINNGEN lookup
Thyroid problem (not cancer)
Hypothyroidism or myxedema
Hypothyroidism/myxoedema | non-cancer illness code, self-reported
Levothyroxine sodium | treatment/medication code
Diabetes or endocrine disease
Hypothyroidism
Hypothyroidism nos
Type 1 diabetes
Hypothyroidism, strict autoimmune, 3 medication purchases required
Hypothyroidism, other/unspecified
Autoimmune diseases
Hypothyroidism, strict autoimmune
Disorders of the thyroid gland
Hyperthyroidism or thyrotoxicosis
Serum total protein level
Hyperthyroidism/thyrotoxicosis | non-cancer illness code, self-reported
Basal cell carcinoma
Hypothyroidism, levothyroxin purchases
Hypothyroidism and >3 levothyroxin purchases
Type 1 diabetes
Type 1 diabetes
Type 1 diabetes
Thyrotoxicosis with diffuse goitre
Hypothyroidism, drug reimbursement
Thyroxine product | treatment/medication code
Thyrotoxicosis
Hypothyroidism (congenital or acquired)
Type 1 diabetes without complications
Disorders of thyroid, IBD co-morbidities
Type 1 diabetes, wide definition, subgroup 1
Thyrotoxicosis, other and/or unspecified
$-\log_{10}(p)$
75
50
25
0
cell proliferation disorder
disease of visual system
endocrine system disease
gastrointestinal disease
immune system disease
infectious disease
injury, poisoning or other complication
integumentary system disease
measurement
musculoskeletal or connective tissue disease
nervous system disease
pancreas disease
phenotype
pregnancy or perinatal disease
reproductive system or breast disease
respiratory or thoracic disease
Uncategorised
Trait category
▲: Positive Beta
▼: Negative Beta

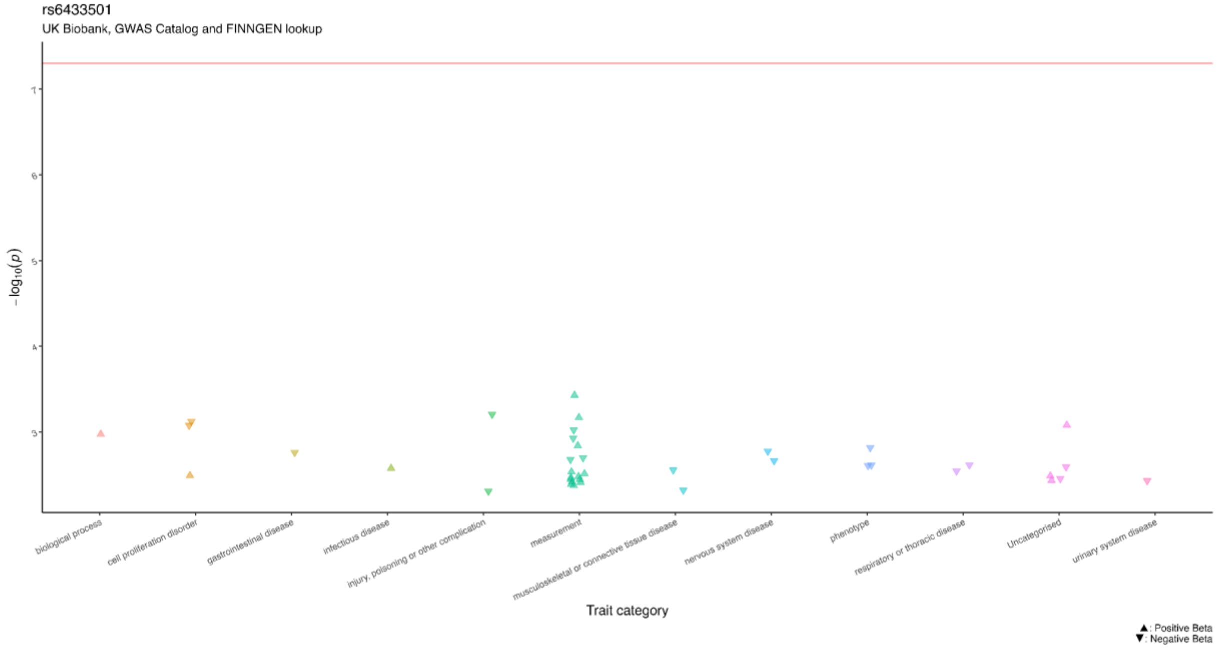
rs6433501
UK Biobank, GWAS Catalog and FINNGEN lookup
$-\log_{10}(p)$
7
6
5
4
3
biological process
cell proliferation disorder
gastrointestinal disease
infectious disease
injury, poisoning or other complication
measurement
musculoskeletal or connective tissue disease
nervous system disease
phenotype
respiratory or thoracic disease
Uncategorised
urinary system disease
Trait category
▲: Positive Beta
▼: Negative Beta

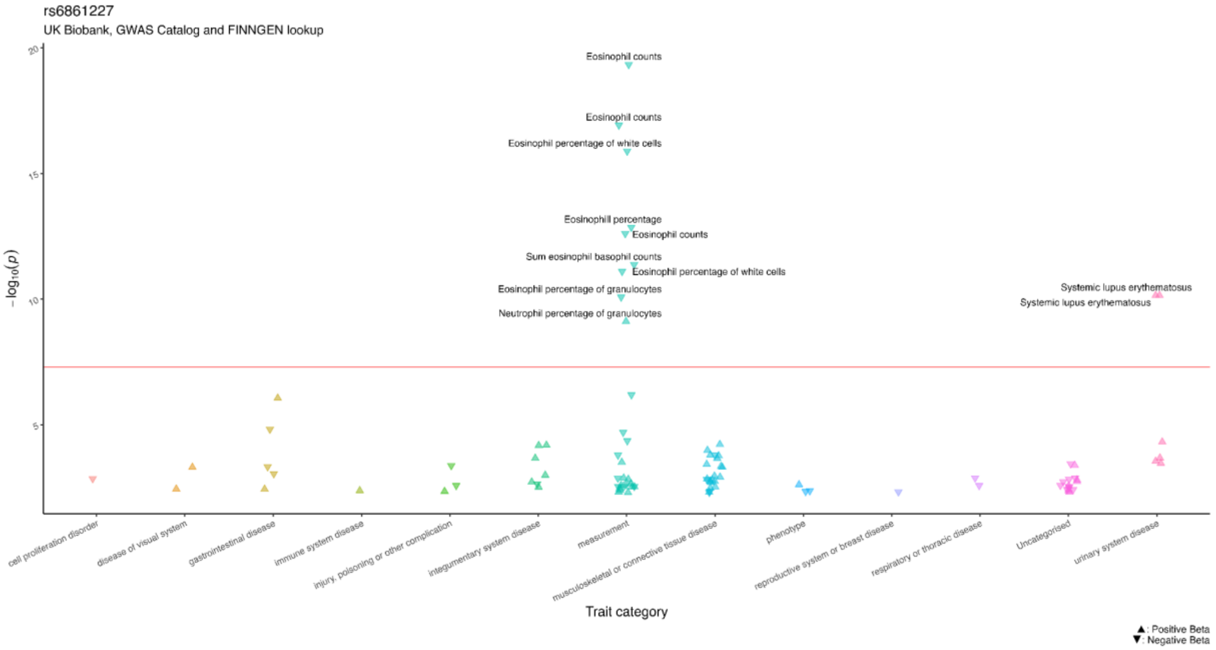
rs6861227
UK Biobank, GWAS Catalog and FINNGEN lookup
$-\log_{10}(p)$
Eosinophil counts
Eosinophil counts
Eosinophil percentage of white cells
Eosinophill percentage
Eosinophil counts
Sum eosinophil basophil counts
Eosinophil percentage of white cells
Eosinophil percentage of granulocytes
Neutrophil percentage of granulocytes
Systemic lupus erythematosus
Systemic lupus erythematosus
cell proliferation disorder
disease of visual system
gastrointestinal disease
immune system disease
injury, poisoning or other complication
integumentary system disease
measurement
musculoskeletal or connective tissue disease
phenotype
reproductive system or breast disease
respiratory or thoracic disease
Uncategorised
urinary system disease
Trait category
▲: Positive Beta
▼: Negative Beta

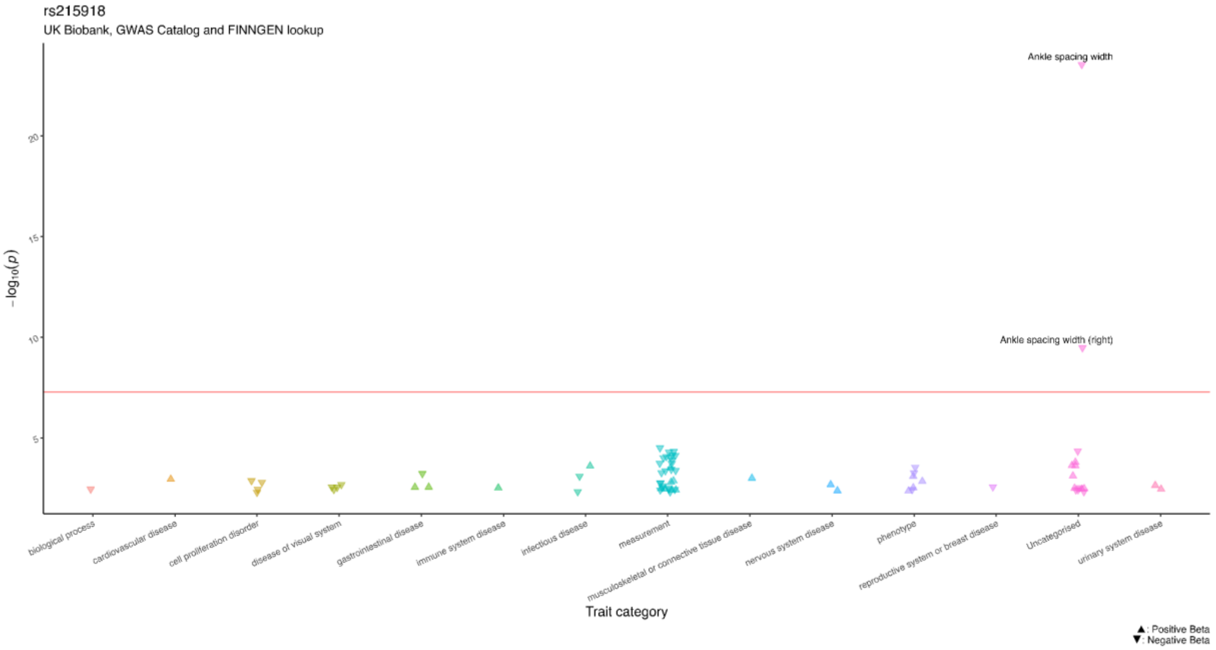
rs215918
UK Biobank, GWAS Catalog and FINNGEN lookup
$-\log_{10}(p)$
Ankle spacing width
Ankle spacing width (right)
biological process
cardiovascular disease
cell proliferation disorder
disease of visual system
gastrointestinal disease
immune system disease
infectious disease
measurement
musculoskeletal or connective tissue disease
nervous system disease
phenotype
reproductive system or breast disease
Uncategorised
urinary system disease
Trait category
▲: Positive Beta
▼: Negative Beta

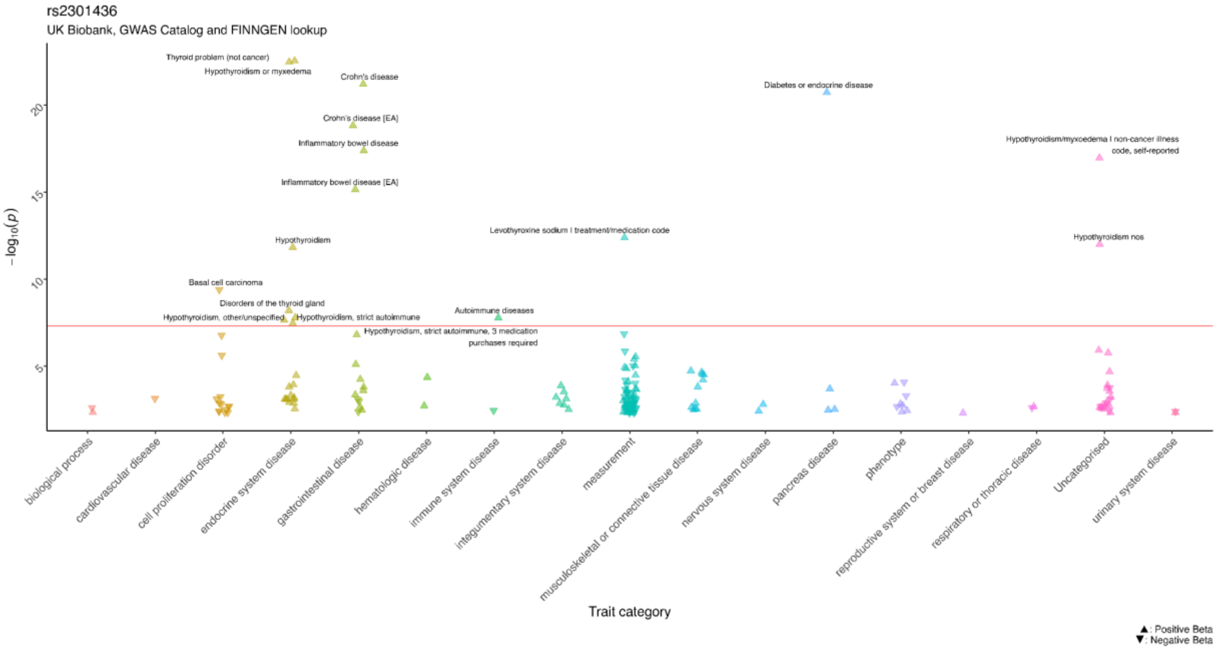

rs2301436
UK Biobank, GWAS Catalog and FINNGEN lookup
Thyroid problem (not cancer)
Hypothyroidism or myxedema
Crohn's disease
Diabetes or endocrine disease
Crohn's disease [EA]
Inflammatory bowel disease
Hypothyroidism/myxoedema | non-cancer illness code, self-reported
Inflammatory bowel disease [EA]
Levothyroxine sodium | treatment/medication code
Hypothyroidism
Hypothyroidism nos
Basal cell carcinoma
Disorders of the thyroid gland
Autoimmune diseases
Hypothyroidism, other/unspecified
Hypothyroidism, strict autoimmune
Hypothyroidism, strict autoimmune, 3 medication purchases required
$-\log_{10}(p)$
biological process
cardiovascular disease
cell proliferation disorder
endocrine system disease
gastrointestinal disease
hematologic disease
immune system disease
integumentary system disease
measurement
musculoskeletal or connective tissue disease
nervous system disease
pancreas disease
phenotype
reproductive system or breast disease
respiratory or thoracic disease
Uncategorised
urinary system disease
Trait category
▲: Positive Beta
▼: Negative Beta

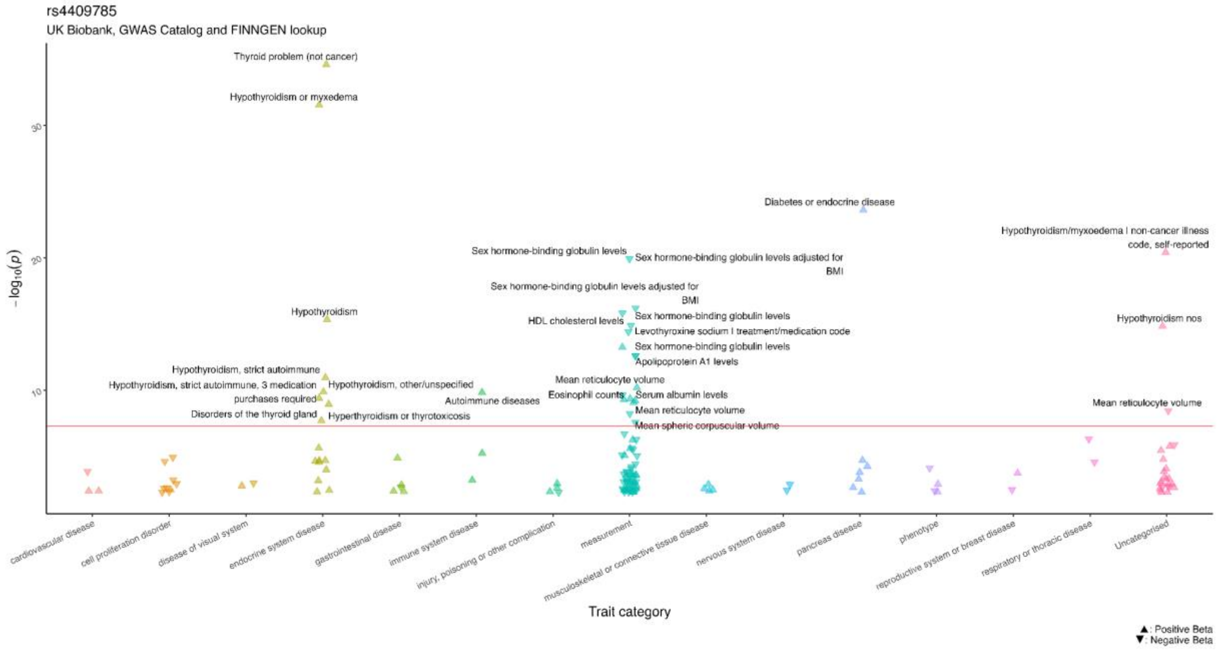

rs4409785
UK Biobank, GWAS Catalog and FINNGEN lookup
Thyroid problem (not cancer)
Hypothyroidism or myxedema
Diabetes or endocrine disease
Hypothyroidism/myxoedema | non-cancer illness code, self-reported
Sex hormone-binding globulin levels
Sex hormone-binding globulin levels adjusted for BMI
Sex hormone-binding globulin levels adjusted for BMI
Hypothyroidism
Sex hormone-binding globulin levels
HDL cholesterol levels
Levothyroxine sodium | treatment/medication code
Hypothyroidism nos
Sex hormone-binding globulin levels
Apolipoprotein A1 levels
Hypothyroidism, strict autoimmune
Hypothyroidism, strict autoimmune, 3 medication purchases required
Hypothyroidism, other/unspecified
Mean reticulocyte volume
Eosinophil counts
Serum albumin levels
Autoimmune diseases
Mean reticulocyte volume
Mean reticulocyte volume
Disorders of the thyroid gland
Hyperthyroidism or thyrotoxicosis
Mean spheric corpuscular volume
$-\log_{10}(p)$
cardiovascular disease
cell proliferation disorder
disease of visual system
endocrine system disease
gastrointestinal disease
immune system disease
injury, poisoning or other complication
measurement
musculoskeletal or connective tissue disease
nervous system disease
pancreas disease
phenotype
reproductive system or breast disease
respiratory or thoracic disease
Uncategorised
Trait category
▲: Positive Beta
▼: Negative Beta

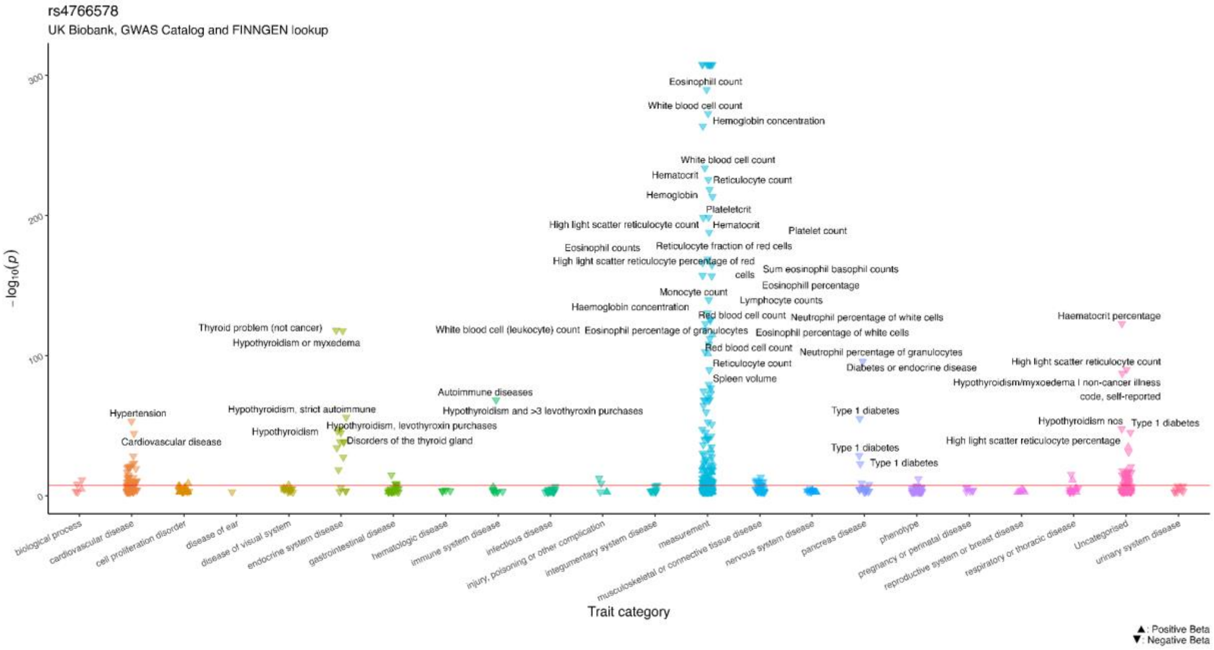
rs4766578
UK Biobank, GWAS Catalog and FINNGEN lookup
Eosinophill count
White blood cell count
Hemoglobin concentration
White blood cell count
Hematocrit
Reticulocyte count
Hemoglobin
Plateletcrit
High light scatter reticulocyte count
Hematocrit
Platelet count
Eosinophil counts
Reticulocyte fraction of red cells
High light scatter reticulocyte percentage of red cells
Sum eosinophil basophil counts
Eosinophill percentage
Monocyte count
Lymphocyte counts
Haemoglobin concentration
Red blood cell count
Neutrophil percentage of white cells
Haematocrit percentage
Thyroid problem (not cancer)
Hypothyroidism or myxedema
White blood cell (leukocyte) count
Eosinophil percentage of granulocytes
Eosinophil percentage of white cells
Red blood cell count
Neutrophil percentage of granulocytes
Reticulocyte count
Diabetes or endocrine disease
High light scatter reticulocyte count
Spleen volume
Hypothyroidism/myxoedema I non-cancer illness code, self-reported
Autoimmune diseases
Hypertension
Hypothyroidism, strict autoimmune
Hypothyroidism and >3 levothyroxin purchases
Type 1 diabetes
Hypothyroidism
Hypothyroidism, levothyroxin purchases
Hypothyroidism nos
Type 1 diabetes
Cardiovascular disease
Disorders of the thyroid gland
High light scatter reticulocyte percentage
Type 1 diabetes
Type 1 diabetes
−log10(p)
0
100
200
300
biological process
cardiovascular disease
cell proliferation disorder
disease of ear
disease of visual system
endocrine system disease
gastrointestinal disease
hematologic disease
immune system disease
infectious disease
injury, poisoning or other complication
integumentary system disease
measurement
musculoskeletal or connective tissue disease
nervous system disease
pancreas disease
phenotype
pregnancy or perinatal disease
reproductive system or breast disease
respiratory or thoracic disease
Uncategorised
urinary system disease
Trait category
▲: Positive Beta
▼: Negative Beta

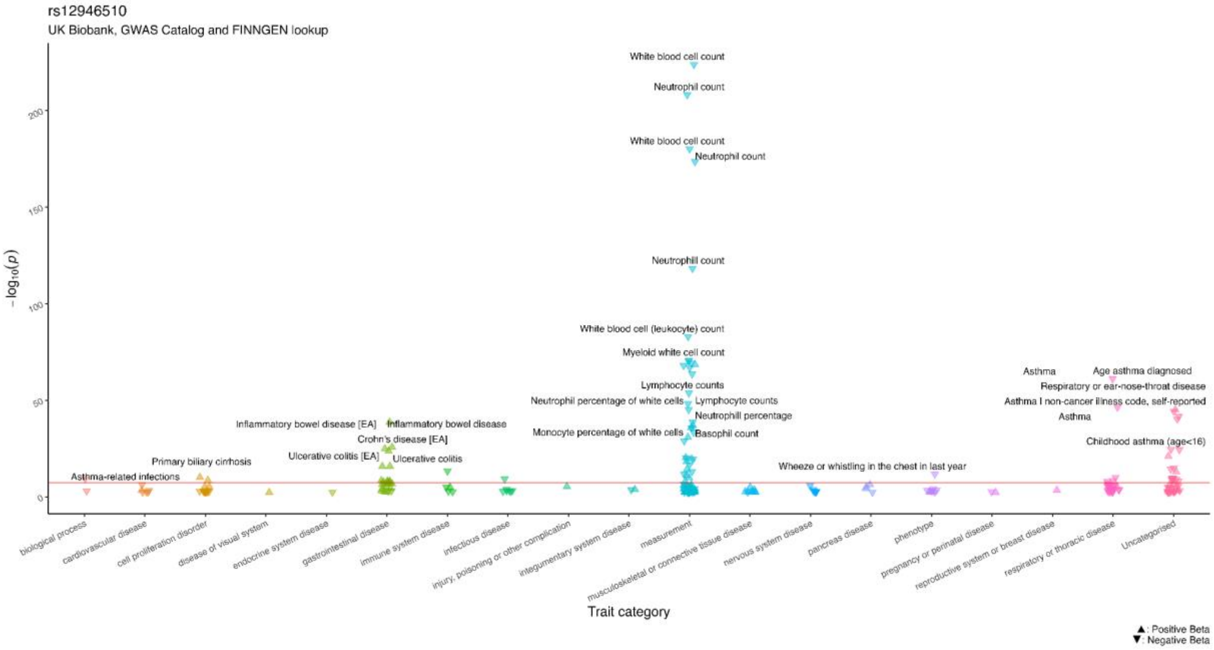
rs12946510
UK Biobank, GWAS Catalog and FINNGEN lookup
White blood cell count
Neutrophil count
White blood cell count
Neutrophil count
Neutrophill count
White blood cell (leukocyte) count
Myeloid white cell count
Asthma
Age asthma diagnosed
Lymphocyte counts
Respiratory or ear-nose-throat disease
Neutrophil percentage of white cells
Lymphocyte counts
Asthma I non-cancer illness code, self-reported
Neutrophill percentage
Asthma
Inflammatory bowel disease [EA]
Inflammatory bowel disease
Monocyte percentage of white cells
Basophill count
Crohn's disease [EA]
Childhood asthma (age<16)
Ulcerative colitis [EA]
Ulcerative colitis
Primary biliary cirrhosis
Wheeze or whistling in the chest in last year
Asthma-related infections
−log10(p)
0
50
100
150
200
biological process
cardiovascular disease
cell proliferation disorder
disease of visual system
endocrine system disease
gastrointestinal disease
immune system disease
infectious disease
injury, poisoning or other complication
integumentary system disease
measurement
musculoskeletal or connective tissue disease
nervous system disease
pancreas disease
phenotype
pregnancy or perinatal disease
reproductive system or breast disease
respiratory or thoracic disease
Uncategorised
Trait category
▲: Positive Beta
▼: Negative Beta

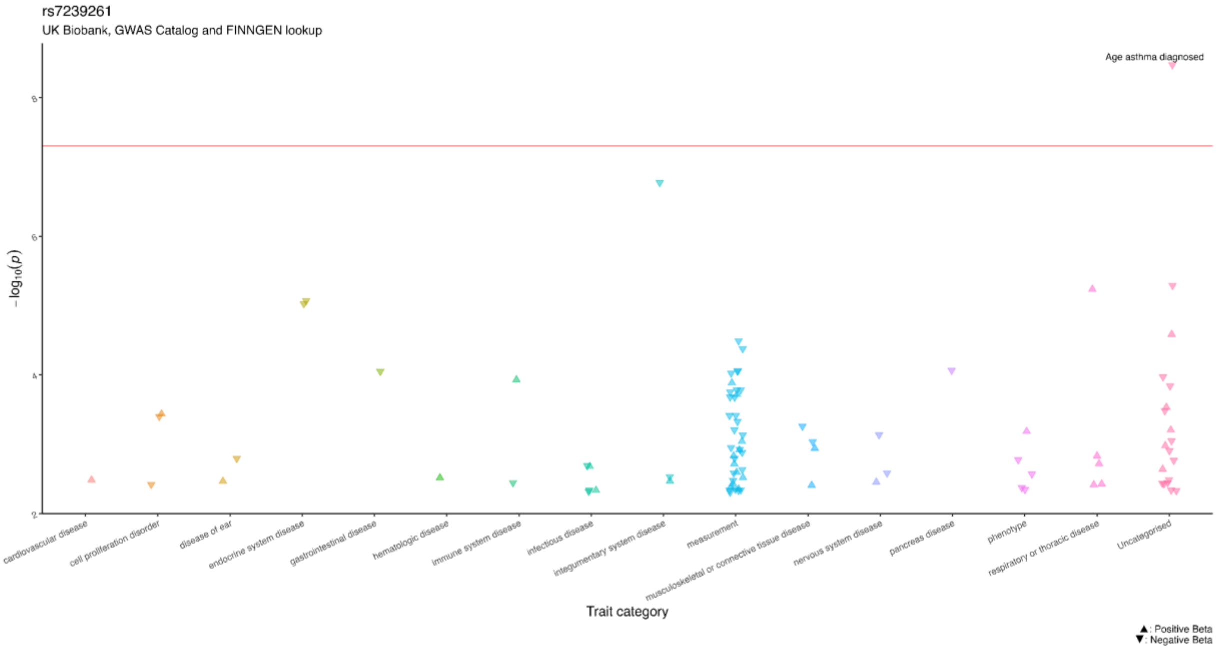

Manhattan plots of all index SNPs, except the MHC, with associations for traits from different data sources aggregated by OpenTargets genetics (Ghoussaini et al., 2021). The datasets include associations identified by the SAIGE study (https://www.leelabsg.org/resources) and the Neale lab (https://www.nealelab.is/uk-biobank/) conducted in the UK Biobank, summary statistics downloaded from NHGRI-EBI GWAS Catalog database GWAS Catalog (https://www.ebi.ac.uk/gwas/), and FinnGen Release 6 (https://r6.finngen.fi/). The y-axis reflect the significance level, the y-axis reflects different trait categories, the red line indicates genome-wide significance ($P < 5e^{-8}$). Genome-wide significant associations are labeled.

Sample description of genotypes

This section includes detailed information on data processing and quality control for all samples we received individual-level genotypes from. For more information on data collection, processing, quality parameters, and imputation of the published cohorts we obtained summary level data please see the respective publications[2,3].

Charité Universitätsmedizin Berlin

Sample ascertainment
All myasthenia gravis patients were enrolled in the study via the myasthenia gravis outpatient clinic of the Charité Universitätsmedizin Berlin Campus Mitte Department of Neurology. All patients met the diagnostic criteria for Myasthenia Gravis according to ICD-10 code G70.0. The control sample was ascertained via the Berlin Psychosis Study and the Berlin Research Initiative for Diagnostics Genetics and Environmental Factors of Schizophrenia. Control subjects had no diagnosis of schizophrenia, schizoaffective disorder, or bipolar disorder. Additionally, the controls were filtered for individuals who self-reported autoimmune or neurological disorders.
Ethics statement
All study participants gave written informed consent and the study received ethical approval from the Charité Universitätsmedizin Berlin's institutional review board under the identifier EA1/281/10.
Materials and methods
Genome-wide genotyping is performed on DNA samples either derived from whole-blood EDTA samples or saliva samples collected using 1.0 ml OraGene (Genotek, Ottawa, Ontario, Canada) saliva DNA-Self-Collection kits. DNA samples were assayed on the Illumina Infinium Global Screening array (GSA) MD BeadChip (Illumina, San Diego, CA) at ERASMUS Medical Center, Human Genotyping Facility (HuGe-F), The Netherlands.

Leiden University Medical Center

Sample ascertainment
Patients were recruited in the myasthenia gravis outpatient clinic at the Leiden University Medical Center (LUMC) and upon consent samples were stored in the LUMC biobank ("Neurologische ziekten"). For this specific study patients were identified from the biobank based on the presence of a positive MuSK antibody test (RSR Ltd) or AChR antibody test (RSR Ltd).
Ethics statement
All included subjects gave permission for DNA analysis. The study was conducted in accordance with the Declaration of Helsinki and was approved by the local medical ethics committee (Protocol number: B19.001).
Materials and methods
All samples were genotyped on the Illumina Infinium Global Screening array (GSA) MD BeadChip (Illumina, San Diego, CA). The 102 MG cases collected at the LUMC were merged with the 372 MG cases and 783 controls collected at CCM. Quality control and imputation were carried out using the RICOPILI pipeline using standard parameters described in detail in the methods section of this publication. 59 controls and 78 cases were excluded during quality control. 6 additional cases were subsequently excluded due to sample overlap with the Gregersen et al., 2012 EOMG sample. Before imputation, the genomic inflation factor λ_{GC} was 1.026. Overall, 390 cases, 724 controls, and 419,097 SNPs passed quality control.

Gregersen et al. 2012

The raw genotypes of 649 early-onset Myasthenia Gravis cases and 2596 controls were shared via the first author. All cases were diagnosed under the age of 50 and tested positive for autoantibodies against the acetylcholine receptor in serum blood. A detailed description of the sample collection procedures is included in the full publication[4]. The sample was genotyped on multiple chips including Illumina 370K, Illumina 610K, Illumina 550K, and Illumina 1Mb. We split the study cohort into three separate samples for middle European, Scandinavian, and English individuals to control population structure. The English cohort included 276 cases and 1169 controls, and 266,934 SNPs with a genomic inflation factor of λ_{GC}=1.036 after quality control. The Scandinavian cohort included 228 cases and 908 controls, and 263,823 SNPs passed quality control with a genomic inflation factor of λ_{GC}=1.071. Finally, the middle European cohort included 136 cases, 490 controls, and 261,207 SNPs were retained after quality control with a genomic inflation factor of λ_{GC}=1.031. Imputation and GWAS were run separately for the 3 individual cohorts.

Seldin et al. 2015

The sample of 320 late-onset myasthenia gravis cases was provided by the last author of the original paper[5]. All cases were diagnosed at the age of 50 or later and tested positive for autoantibodies against the acetylcholine receptor in serum blood. The cases were merged with a control sample downloaded via the dbGAP accession code phs000882.v1.p1. Both case and control samples were genotyped on Illumina's HumanOmni 2.5 chips. Cases and controls were matched by PCA. Overall, 243 cases and 481 controls were left after quality control and matching. However, due to a large sample overlap with the LUMC cohort another 154 genetically identical cases were excluded from the sample. After final sample exclusion 89 cases, 481 controls, and 266566 SNPs with a genomic inflation factor of λ_{GC}=1.022 were retained for imputation after quality control, PCA matching, and exclusion of identical individuals.

Renton et al. 2015

We obtained the sample analyzed within the Renton et al. 2015 GWAS study[6] via the dbGAP accession code phs000726.v1.p1 and merged with controls from dbGAP accession code phs000196.v3.p1. The control subjects have no diagnosis of Alzheimer's disease, Bipolar disorder, Multiple Sclerosis, Amyotrophic Lateral Sclerosis, Ataxia, Dystonia, Parkinson's disease, Autism, Dementia, Epilepsy, or Schizophrenia. A detailed description of the sample collection procedures is included in respective publications. The cases were genotyped on the Illumina HumanOmniExpress Beadchip and the controls on HumanOmni1-Quad BeadChip. Quality control and imputation were carried out using the RICOPILI pipeline using standard parameters described in detail in the methods section of this publication. Overall, 154 cases and 209 controls were filtered out during quality control. 808 cases, 1,777 controls, and 571,934 SNPs passed quality control. The Renton et al. 2015 sample is included in the larger Chia et al. 2022 publication[2]. Phenotypic information on the exact age of onset and sample donation for both cases and controls were available. As these raw phenotypes were not available for the Chia et al. 2022 cort, which was split by age at 60 and not 50 for onset-specific analyses, we used the Renton et al. 2025 cohort in these analyses. We further used the Renton et al., 2015 sample for all analyses that require raw genotypes like HLA imputation.

Sample description of summary-level data

23andMe,Inc.

Ethics statement
Participants provided informed consent and volunteered to participate in the research online, under a protocol approved by the external AAHRPP-accredited IRB, Ethical & Independent (E&I) Review Services. As of 2022, E&I Review Services is part of Salus IRB (https://www.versiticlinicaltrials.org/salusirb). The variant-level data for the 23andMe replication dataset are fully disclosed in the manuscript. Individual-level data are not publicly available due to participant confidentiality, and in accordance with the IRB-approved protocol under which the study was conducted.

BioVU

Ethics statement
The BioVU[7] is the Vanderbilt University' biorepository which contains de-identified samples obtained from unused blood collected during regular clinical testing. As of January 2023, 300,000 DNA samples have been included in BioVU. Vanderbilt clinical patients have to sign a consent form if they wish to donate excess blood samples to the BioVU biorepository (https://victr.vumc.org/biovu-consent/). The project was approved by Vanderbilt's Institutional Review Board (IRB) (#190418). For more information on BioVU data collection and processing please visit https://victr.vumc.org/what-is-biovu/
Materials and methods
Myasthenia gravis cases were identified based on at least one instance of the ICD-10 code 70.0 or the ICD-9 code 358.0 in their electronic medical record. Controls were randomly matched with cases based on a five-to-one ratio and association analysis of genotyped common variants/SNPs with a MAF >1% was carried out via logistic regression implemented in PLINK 2.0[8].

deCODE Genetics/ Amgen, Inc.

Ethics statement
The study was approved by the National Bioethics Committee (Approval no. VSN-16-042) following review by the Icelandic Data Protection Authority (DPA). Participants signed an informed consent prior to donating a blood or buccal sample. All personal identifiers of the participants' data were encrypted by a third-party system, approved and monitored by the DPA, and all data processing complies with the instructions of the DPA (PV_2017060950ÞS).
Study sample
Information of individuals diagnosed with myasthenia gravis with ICD-10 code G70.0 or ICD-9 code 358.0 was obtained from Landspitali, the National Hospital of Iceland (from 1988-2022) and from the Directorate of Health (Register of Contacts with Medical Specialists in Private Practice, from 2012-2019, and Register of Primary Health Care Contacts, from 2004-2019.
Genotyping and imputation
Over 166,000 Icelanders have been genotyped using various Illumina single-nucleotide polymorphism (SNP) chips and their genotypes phased using long-range phasing which allows for improving genotype calls using the information about haplotype sharing[9]. Of those 166,000 Icelanders, over 49,000 have been whole-genome sequenced using GAIIx, HiSeq, HiSeqX, and NovaSeq Illumina technology to a mean depth of at least 17.8×[10]. Single nucleotide polymorphisms (SNPs) and indels were identified and their genotypes were called using joint calling with Graphtyper[11]. Subsequently, 37.6 million high-quality sequence variants were imputed into the 166,000 genotyped Icelanders, as well as their first- and second-degree relatives using genealogical information to increase the sample size and power for association analysis[12].
Association analysis
We applied logistic regression, assuming an additive model, to test for association between sequence variants and Myasthenia gravis[10]. Adjustments were made for sex, current age or age at death, country of origin, blood sample availability for the individual and an indicator function for the overlap of the lifetime of the individual with the time span of phenotype collection[13].

Estonian Biobank

Ethics statement

Individual level data analysis in EstBB was carried out under ethical approval 1.1-12/624 from the Estonian Committee on Bioethics and Human Research (Estonian Ministry of Social Affairs). All biobank participants have signed a broad informed consent form.

Materials and methods

The Estonian Biobank (EstBB) is a population-based biobank with 200k participants. The 198k data freeze was used for the analyses described here. Participants with myasthenia gravis were identified using the ICD-10 code system (information on ICD codes is obtained via linking with the national Health Insurance Fund and other databases (Leitsalu, L. et al. Cohort profile: Estonian biobank of the Estonian Genome Center, University of Tartu. Int. J. Epidemiol. 44, 1137–1147 (2015).) Individuals with Myasthenia gravis were identified using the ICD-10 code G70.0. Two separate cohorts were generated: first, with Myasthenia gravis onset over 50 yo (n=105, controls n=521); second, onset under 50 yo (n=101, controls n=534). GWAS was carried out using REGENIE (v2.2.4). All EstBB participants have been genotyped at the Core Genotyping Lab of the Institute of Genomics, University of Tartu, using Illumina Global Screening Array v1.0 and v2.0. Samples were genotyped and PLINK format files were created using Illumina GenomeStudio v2.0.4. Individuals were excluded from the analysis if their call-rate was <95% or if sex defined based on heterozygosity of X chromosome did not match sex in phenotype data. Before imputation, variants were filtered by call-rate <95%, HWE p-value < 1e-4 (autosomal variants only), and minor allele frequency <1%. Variant positions were updated to b37 and all variants were changed to be from TOP strand using GSAMD-24v1-0_20011747_A1-b37.strand.RefAlt.zip files from https://www.well.ox.ac.uk/~wrayner/strand/ webpage. Prephasing was done using Eagle v2.3 software[14] (number of conditioning haplotypes Eagle2 uses when phasing each sample was set to:–Kpbwt=20000) and imputation was done using Beagle v.28 Sep18.793[15] with effective population size ne = 20,000. Population specific imputation reference panel of 2297 WGS samples was used[16]. (Genotype and imputation information from: Laisk, T., Lepamets, M., Koel, M. et al. Genome-wide association study identifies five risk loci for pernicious anemia. Nat Commun 12, 3761 (2021). Computations were performed in the High Performance Computing Center, University of Tartu. The activities of the EstBB are regulated by the Human Genes Research Act, which was adopted in 2000 specifically for the operations of EstBB.

FinnGen

Ethics statement and materials and methods

Patients and control subjects in FinnGen provided informed consent for biobank research, based on the Finnish Biobank Act. Alternatively, separate research cohorts, collected prior the Finnish Biobank Act came into effect (in September 2013) and start of FinnGen (August 2017), were collected based on study-specific consents and later transferred to the Finnish biobanks after approval by Fimea (Finnish Medicines Agency), the National Supervisory Authority for Welfare and Health. Recruitment protocols followed the biobank protocols approved by Fimea. The Coordinating Ethics Committee of the Hospital District of Helsinki and Uusimaa (HUS) statement number for the FinnGen study is Nr HUS/990/2017.

FinnGen is a pre-competitive partnership of Finnish biobanks and their background organizations (universities and university hospitals) and international pharmaceutical industry partners and Finnish biobank cooperative (FINBB). All FinnGen partners are listed here: https://www.finngen.fi/en/partners. The FinnGen study is approved by Finnish Institute for Health and Welfare (permit numbers: THL/2031/6.02.00/2017, THL/1101/5.05.00/2017, THL/341/6.02.00/2018, THL/2222/6.02.00/2018, THL/283/6.02.00/2019, THL/1721/5.05.00/2019 and THL/1524/5.05.00/2020), Digital and population data service agency (permit numbers: VRK43431/2017-3, VRK/6909/2018-3, VRK/4415/2019-3), the Social Insurance Institution (permit numbers: KELA 58/522/2017, KELA 131/522/2018, KELA 70/522/2019, KELA 98/522/2019, KELA 134/522/2019, KELA 138/522/2019, KELA 2/522/2020, KELA 16/522/2020), Findata permit numbers THL/2364/14.02/2020, THL/4055/14.06.00/2020, THL/3433/14.06.00/2020, THL/4432/14.06/2020, THL/5189/14.06/2020, THL/5894/14.06.00/2020, THL/6619/14.06.00/2020, THL/209/14.06.00/2021, THL/688/14.06.00/2021, THL/1284/14.06.00/2021, THL/1965/14.06.00/2021, THL/5546/14.02.00/2020 and Statistics Finland (permit numbers: TK-53-1041-17 and TK/143/07.03.00/2020 (earlier TK-53-90-20)).

The Biobank Access Decisions for FinnGen samples and data utilized in FinnGen Data Freeze 7 include: THL Biobank BB2017_55, BB2017_111, BB2018_19, BB_2018_34, BB_2018_67, BB2018_71, BB2019_7, BB2019_8, BB2019_26, BB2020_1, Finnish Red Cross Blood Service Biobank 7.12.2017, Helsinki Biobank HUS/359/2017, Auria Biobank AB17-5154 and amendment #1 (August 17 2020), Biobank Borealis of Northern Finland_2017_1013, Biobank of Eastern Finland 1186/2018 and amendment 22 § /2020, Finnish Clinical Biobank Tampere MH0004 and amendments (21.02.2020 & 06.10.2020), Central Finland Biobank 1-2017, and Terveystalo Biobank STB 2018001.

Million Veteran Program

The Million Veteran Program (MVP) is a national research program to learn how genes, lifestyle, and military exposures affect health and illness. Envisioned as a US Veterans Affairs (VA)-based mega-biobank, the MVP was launched to establish a national, representative, and longitudinal study of Veterans for genomic (and non-genomic) research that combines data from survey instruments, the electronic health record, and biospecimens. Since launching in 2011, over 825,000 Veteran partners have joined one of the world's largest programs on genetics and health.

We used data release version 4 of the MVP[17]. Linked and de-identified electronic health records (EHRs) were queried using the Veterans Affairs Informatics and Computing Infrastructure to identify individuals with International Classification of Disease (ICD) codes for MG. The range of diagnosis dates was between May 1992 and December 2019. Identified cases were matched to controls based on age, sex, and the first three principal components on a one-to-five ratio. GWAS was performed in PLINK 2.0 against dosage data using the glm function on case versus control status in unrelated individuals as outcome and age, sex, and the first ten principal components as covariates. Alleles with MAF <1%, genotype missingness >%20, and with Hardy-Weinberg equilibrium (HWE) p-values less than 5 x 10-5 were excluded.

The Central Veterans Affairs Institutional Review Board (IRB) and site-specific IRBs in West Haven, CT and San Diego, CA approved the MVP study.

UK Biobank

Ethics statement

For detailed information on the UK Biobank's ethics declaration please visit the following link: https://www.ukbiobank.ac.uk/learn-more-about-uk-biobank/about-us/ethics. UK Biobank is a large-scale biomedical database and research resource containing genetic, lifestyle and health information from half a million UK participants. UK Biobank's database, which includes blood samples, heart and brain scans and genetic data of the 500,000 volunteer participants, is globally accessible to approved researchers who are undertaking health-related research that's in the public interest. UK Biobank recruited 500,000 people aged between 40-69 years in 2006-2010 from across the UK. *Materials and methods*

With their consent, they provided detailed information about their lifestyle, physical measures and had blood, urine and saliva samples collected and stored for future analysis. UK Biobank's research resource is a major contributor in the advancement of modern medicine and treatment, enabling better understanding of the prevention, diagnosis and treatment of a wide range of serious and life-threatening illnesses – including cancer, heart diseases and stroke. Since the UK Biobank resource was opened for research use in April 2012, over 20,000 researchers from 90+ countries have been approved to use it and more than 2,000 peer-reviewed papers that used the resource have now been published. UK Biobank is generously supported by its founding funders the Wellcome Trust and UK Medical Research Council, as well as the British Heart Foundation, Cancer Research UK, Department of Health, Northwest Regional Development Agency and Scottish Government. The organization has over 150 dedicated members of staff, based in multiple locations across the UK. You can find out more about UK Biobank at http://www.ukbiobank.ac.uk. Quality checked and imputed genotypes of UK biobank participants were accessed via an analysis pipeline on the LISA, SURFsara computer cluster through the Project Number: 16406. Cases were intensified via the ICD-10 code G70.0 in the data field Data-Field 41270 (Diagnoses - ICD10, Category: Health-related outcomes, Hospital inpatient, Summary Diagnoses, Health outcomes). Cases and controls were matched by age, sex, and the first four principal components in a one-to-three ratio. The genome-wide association study of SNPs with a MAF >1% was conducted using the logistic regression model implemented in PLINK 2.0[8] with case versus controls status as the outcome and sex, genotyping array, age, and the first principal components associated with the ancestry as covariates.

Supplementary references

1. Ghoussaini M, Mountjoy E, Carmona M, et al. Open Targets Genetics: systematic identification of trait-associated genes using large-scale genetics and functional genomics. *Nucleic Acids Res.* 2021;49(D1):D1311-D1320. doi:10.1093/nar/gkaa840
2. Lam M, Awasthi S, Watson HJ, et al. RICOPILI: Rapid Imputation for COnsortias PIpeLIne. *Bioinformatics*. 2020;36(3):930-933. doi:10.1093/bioinformatics/btz633
3. Chia R, Saez-Atienzar S, Murphy N, et al. Identification of genetic risk loci and prioritization of genes and pathways for myasthenia gravis: a genome-wide association study. *Proc Natl Acad Sci U S A*. 2022;119(5):e2108672119. doi:10.1073/pnas.2108672119
4. Topaloudi A, Zagoriti Z, Flint AC, et al. Myasthenia gravis genome-wide association study implicates AGRN as a risk locus. *J Med Genet*. 2022;59(8):801-809. doi:10.1136/jmedgenet-2021-107953
5. Gregersen PK, Kosoy R, Lee AT, et al. Risk for myasthenia gravis maps to a (151) Pro→Ala change in TNIP1 and to human leukocyte antigen-B*08. *Ann Neurol*. 2012;72(6):927-935. doi:10.1002/ana.23691
6. Seldin MF, Alkhairy OK, Lee AT, et al. Genome-Wide Association Study of Late-Onset Myasthenia Gravis: Confirmation of TNFRSF11A and Identification of ZBTB10 and Three Distinct HLA Associations. *Mol Med*. 2015;21(1):769-781. doi:10.2119/molmed.2015.00232
7. Renton AE, Pliner HA, Provenzano C, et al. A genome-wide association study of myasthenia gravis. *JAMA Neurol*. 2015;72(4):396-404. doi:10.1001/jamaneurol.2014.4103
8. Roden D, Pulley J, Basford M, et al. Development of a Large-Scale De-Identified DNA Biobank to Enable Personalized Medicine. *Clin Pharmacol Ther*. 2008;84(3):362-369. doi:10.1038/clpt.2008.89
9. Chang CC, Chow CC, Tellier LC, Vattikuti S, Purcell SM, Lee JJ. Second-generation PLINK: rising to the challenge of larger and richer datasets. *GigaScience*. 2015;4(1):7. doi:10.1186/s13742-015-0047-8
10. Kong A, Masson G, Frigge ML, et al. Detection of sharing by descent, long-range phasing and haplotype imputation. *Nat Genet*. 2008;40(9):1068-1075. doi:10.1038/ng.216
11. Gudbjartsson DF, Helgason H, Gudjonsson SA, et al. Large-scale whole-genome sequencing of the Icelandic population. *Nat Genet*. 2015;47(5):435-444. doi:10.1038/ng.3247
12. Eggertsson HP, Jonsson H, Kristmundsdottir S, et al. Graphtyper enables population-scale genotyping using pangenome graphs. *Nat Genet*. 2017;49(11):1654-1660. doi:10.1038/ng.3964
13. Gudbjartsson DF, Sulem P, Helgason H, et al. Sequence variants from whole genome sequencing a large group of Icelanders. *Sci Data*. 2015;2(1):150011. doi:10.1038/sdata.2015.11
14. Gudbjartsson DF, Sulem P, Helgason H, et al. Sequence variants from whole genome sequencing a large group of Icelanders. *Sci Data*. 2015;2(1):150011. doi:10.1038/sdata.2015.11
15. Loh PR, Danecek P, Palamara PF, et al. Reference-based phasing using the Haplotype Reference Consortium panel. *Nat Genet*. 2016;48(11):1443-1448. doi:10.1038/ng.3679
16. Browning SR, Browning BL. Rapid and Accurate Haplotype Phasing and Missing-Data Inference for Whole-Genome Association Studies By Use of Localized Haplotype Clustering. *Am J Hum Genet*. 2007;81(5):1084-1097.
17. Mitt M, Kals M, Pärn K, et al. Improved imputation accuracy of rare and low-frequency variants using population-specific high-coverage WGS-based imputation reference panel. *Eur J Hum Genet*. 2017;25(7):869-876. doi:10.1038/ejhg.2017.51
18. Gaziano JM, Concato J, Brophy M, et al. Million Veteran Program: A mega-biobank to study genetic influences on health and disease. *J Clin Epidemiol*. 2016;70:214-223. doi:10.1016/j.jclinepi.2015.09.016
