# Supplementary material for: Genome-wide meta-analysis of myasthenia gravis uncovers new loci and provides insights into polygenic prediction
Source: Nat Commun. 2024 Nov 13;15:9839. doi: 10.1038/s41467-024-53595-6 (PMC11560923; doi:10.1038/s41467-024-53595-6)
Supplement: Supplementary file 3 — Description of Additional Supplementary Files [file 41467_2024_53595_MOESM3_ESM.pdf]

Description of Additional Supplementary Files

File Name: Supplementary Data 1
Description: MG discovery GWAS summary statistics for SNPs with p<1e-4 from an inverse-variance-weighted fixed-effects meta-analysis

SNP	name of variant
CHR	chromosome
BP	base pair position (hg19)
P	*P*-value
OR	odds ratio
SE	standard error
A1/A2	allele 1 and allele 2
FRQ_A	frequency of allele1 in affected cases
FRQ_U	frequency of allele1 in unaffected controls
INFO	imputation info score
ngt	number of studies in which this variant was genotyped (vs. imputed)
LD-friends(0.1).p1	list of all variants with LD-r2 > 0.1 to index SNP, in brackets LD-r2 and distance in kb sorted by LD-r2
range.left	left margin of region (defined by LD friends)
range.right	right margin of region (defined by LD friends)
span(kb)	right margin - left margin (in kb)
LD-friends(0.6).p	*range.left.6, range.right.6, and span.6(kb)*as before but with LD-r2 of 0.6 gwas_catalog_span.6
genes.6.50kb(dist2index)	list of genes within the region of friends.6 (±50 kb), in brackets distance to index SNP in kb.

File Name: Supplementary Data 2
Description: EOMG discovery GWAS summary statistics for SNPs with p<1e-4 from an inverse-variance-weighted fixed-effects meta-analysis

See legend of Supplementary Data 1.

File Name: Supplementary Data 3
Description: LOMG discovery GWAS summary statistics for SNPs with p<1e-4 from an inverse-variance-weighted fixed-effects meta-analysis

See legend of Supplementary Data 1.

File Name: Supplementary Data 4

Description: HLA alleles associated with MG from an inverse-variance-weighted fixed-effects meta-analysis

HLA allele	The specific HLA allele being analyzed.
BP	The base pair position of the allele on the chromosome.
Frq cases	Frequency of the allele in individuals with the condition (cases).
Frq controls	Frequency of the allele in individuals without the condition (controls).
OR	Odds Ratio, representing the likelihood of association between the allele and the condition.
SE	Standard Error of the Odds Ratio, indicating the variability of the estimate.
P	P-value, representing the statistical significance of the association between the allele and the condition.

File Name: Supplementary Data 5
Description: HLA alleles associated with EOMG from an inverse-variance-weighted fixed-effects meta-analysis

See legend of Supplementary Data 4.

File Name: Supplementary Data 6
Description: HLA alleles associated with LOMG from an inverse-variance-weighted fixed-effects meta-analysis

See legend of Supplementary Data 4.

File Name: Supplementary Data 7
Description: C4 association analysis results from an inverse-variance-weighted fixed-effects meta-analysis

Phenotype	The specific phenotype being studied (Early onset myasthenia gravis; EOMG, Late onset; LOMG, or combined; MG).
C4 gene composition	The composition or variant of the C4 gene associated with the phenotype.
OR	Odds Ratio, representing the likelihood of association between the C4 gene composition and the phenotype.
95% CI_Lower	The lower bound of the 95% Confidence Interval for the Odds Ratio, indicating the range within which the true effect size is likely to fall.
95% CI_Upper	The upper bound of the 95% Confidence Interval for the Odds Ratio.
P-value	The statistical significance of the association between the C4 gene composition and the phenotype.

File Name: Supplementary Data 8
Description: FUMA prioritized genes within RICOPILI locus boundaries

ensg	ENSG ID
symbol	Gene Symbol
chr	chromosome
start	Starting position of the gene
end	Ending position of the gene
strand	Strand of the gene
type	Gene biotype from Ensembl
entrezID	*entrez ID (if available)*
HUGO	*HUGO (HGNC) gene symbol*
pLI	*pLI score from ExAC database. The probability of being loss-of-function intolerant. The higher the score is, the more intolerant to loss-of-function mutations the gene is.*
ncRVIS	*Non-coding residual variation intolerance score. The higher the score is, the more intolerant to non-coding variation the gene is.*
posMapSNPs (posMap)	*Number of SNPs mapped to gene based on positional mapping (after functional filtering if parameters are given).*
posMapMaxCADD (posMap)	*The maximum CADD score of mapped SNPs by positional mapping.*
eqtlMapSNPs (eqtlMap)	*Number of SNPs mapped to the gene based on eQTL mapping.*
eqtlMapminP (eqtlMap)	*The minimum eQTL P-value of mapped SNPs.*
eqtlMapminQ (eqtlMap)	*The minimum eQTL FDR of mapped SNPs.*
eqtlMapts (eqtlMap)	Tissue types of mapped eQTL SNPs.
eqtlDirection (eqtlMap)	Consequential direction of mapped eQTL SNPs after aligning risk increasing alleles in GWAS and tested alleles in eQTL data source.
ciMap (ciMap)	Yes, if the gene is mapped by chromatin interaction mapping, "No" otherwise.
ciMapts (ciMap)	Tissue/cell types of mapped chromatin interactions.
minGwasP	The minimum P-value of mapped SNPs.
IndSigSNPs	rsID of the independent significant SNPs that are in LD with the mapped SNPs.
GenomicLocus	Index of genomic loci where mapped SNPs are from. Multiple loci can be assigned with ":" delimiter.

File Name: Supplementary Data 9
Description: FUMA annotated variants across credible sets

SNP	name of variant
CHR	chromosome
BP	base pair position (hg19)
pip	Posterior inclusion probability
uniqID	CHR:Position
gene	ENSG ID
nearestGene	The nearest Gene of the SNP based on ANNOVAR annotations. Note that ANNOVAR annotates "consequence" function by prioritising the most deleterious annotation for SNPs which are locating a genomic region where multiple genes are overlapped. Genes are encoded in symbol, if it is available, otherwise Ensembl ID.
dist	Distance to the nearest gene. SNPs which are locating in the gene body or 1kb up- or down-stream of TSS or TES have 0.
func	Functional consequence of the SNP on the gene obtained from ANNOVAR. For exonic SNPs, detail annotation (e.g. non-synonymous, stop gain and so on) is available in ANNOVAR table (annov.txt).
CADD	CADD score which is computed based on 63 annotations. 'NA' if not available.
RDB	RegulomeDB score which is the categorical score (from 1a to 7). 1a is the highest score that the SNP has the most biological evidence to be regulatory element.
minChrState	The minimum 15-core chromatin state across 127 tissue/cell type.
commonChrState	The most common 15-core chromatin state across 127 tissue/cell types.
posMapFilt	Whether the SNP was used for positional mapping or not. 1 is used, otherwise 0. When positional mapping is not performed, all SNPs have 0.
eqtlMapFilt	Whether the SNP was used for eQTL mapping or not. 1 is used, otherwise 0. When eQTL mapping is not performed, all SNPs have 0.
ciMapFilt	Whether the SNP was used for chromatin interaction mapping or not. 1 is used, otherwise 0. When chromatin interaction mapping is not performed, all SNPs have 0.

File Name: Supplementary Data 10
Description: Transcriptome-wide association study, permutation, and co-localization tests, permutation, and co-localization tests results p<1e-4

PANEL	The GTEx panel used
FILE	Full path to the reference weight file used
ID	Feature/gene identifier, taken from --weights file
CHR	Chromosome
P0	Gene start (from --weights)
P1	Gene end (from --weights)
HSQ	Heritability of the gene
BEST.GWAS.ID	rsID of the most significant GWAS SNP in locus
BEST.GWAS.Z	Z-score of the most significant GWAS SNP in locus
EQTL.ID	rsID of the best eQTL in the locus
EQTL.R2	cross-validation R2 of the best eQTL in the locus
EQTL.Z	Z-score of the best eQTL in the locus
EQTL.GWAS.Z	GWAS Z-score for this eQTL
NSNP	Number of SNPs in the locus
MODEL	Best performing model
MODELCV.R2	cross-validation R2 of the best performing model
MODELCV.PV	cross-validation P-value of the best performing model
TWAS.Z	TWAS Z-score (our primary statistic of interest)
TWAS.P	TWAS P-value

File Name: Supplementary Data 11
Description: MG-PRS performance combined MG target sample scored

PT	P-value threshold
N	Sample Size
Propcase	Population prevlanece of cases
NKr2	Nagelkerke's Pseudo R2
pval	P-value
PopRisk	Population Risk
h2l_r2n	Proportion of variance explained on the liability scale
se_h2l_r2	Standard Error of the proportion of variance explained on the liability scale
AUC	Area Under the Curve, representing model performance
OR10decile	Odds Ratio for the 10th Decile
ORL95	Odds Ratio Lower 95% Confidence Interval
ORH95	Odds Ratio Upper 95% Confidence Interval
Ncase	Number of Cases
Ncontrol	Number of Controls
Coeff_with_cov	Coefficient with Covariates Included

File Name: Supplementary Data 12
Description: MG-PRS performance in EOMG target sample derived from logistic regression models

See legend of Supplementary Data 11.

File Name: Supplementary Data 13
Description: MG-PRS performance in LOMG target sample derived from logistic regression models

See legend of Supplementary Data 11.

File Name: Supplementary Data 14
Description: MG-PRS performance in AChR negative target sample derived from logistic regression models

See legend of Supplementary Data 11.

File Name: Supplementary Data 15
Description: MG-PRS performance in AChR positive target sample derived from logistic regression models

See legend of Supplementary Data 11.

File Name: Supplementary Data 16
Description: Genetic correlation of MG with other complex traits via linkage disequilibrium score regression

Trait	"Trait 2", for which the genetic correlation with MG was calculated.
Publication	The original publication of the GWAS summary statistics
N	The sample size of the summary statistics used
rg	Genetic correlation
se	Standard error of rg
p	P-value for rg
h2_obs	Observed scale heritability for Trait 2
h2_obs_se	Standard error of observed scale heritability for Trait 2
h2_int	Single-trait LD Score regression intercept for Trait 2
h2_int_se	Standard error of the single-trait LD Score regression intercept for Trait 2
gcov_int	Cross-trait LD Score regression intercept
gcov_int_se	Standard error of the cross-trait LD Score regression intercept

File Name: Supplementary Data 17
Description: Genetic correlation of EOMG with other complex traits via linkage disequilibrium score regression

See legend of Supplementary Data 16.

File Name: Supplementary Data 18
Description: Genetic correlation of LOMG with other complex traits via linkage disequilibrium score regression

See legend of Supplementary Data 16.

File Name: Supplementary Data 19
Description: Genetic correlation of MG with medical endpoints in FinnGen R8 via linkage disequilibrium score regression p<5e-2

LONGNAME	Long name of the phenotype if applicable
Phenotypes	The phenotype ("Trait 2"), for which the genetic correlation with MG was calculated.
Category	International Classification of Diseases chapter if applicable
n_cases	Sample size of cases
n_control	Sample size of controls
sample_size	FinnGen dataset sample size
rg	Genetic correlation
se	Standard error of rg
p	P-value for rg
h2_obs	Observed scale heritability for Trait 2
h2_obs_se	Standard error of observed scale heritability for Trait 2
h2_int	Single-trait LD Score regression intercept for Trait 2
h2_int_se	Standard error of the single-trait LD Score regression intercept for Trait 2
gcov_int	Cross-trait LD Score regression intercept
gcov_int_se	Standard error of the cross-trait LD Score regression intercept

File Name: Supplementary Data 20
Description: Sample overview for MG datasets

Dataset	The name of the specific dataset used.
N cases	The number of cases included in the analysis.
N controls	The number of control subjects included in the analysis.
PubMed ID	The identifier for the associated publication in PubMed, if applicable.
Data source	The source from which the data was obtained (individual-level genotypes or summary statistics).
Phenotype	Indicates whether the included case phenotype was based on a specific antibody profile and/or ICD-Code.

File Name: Supplementary Data 21
Description: Sample overview EOMG datasets

See legend of Supplementary Data 20.

File Name: Supplementary Data 22
Description: Sample overview LOMG datasets

See legend of Supplementary Data 20.

File Name: Supplementary Data 23
Description: Neurological and autoimmune-related ICD-9 and ICD-10 codes used to exclude control subjects

Diagnosis	The medical diagnosis being referred to.
ICD-10 Code	The International Classification of Diseases, 10th Revision code.
ICD-9 Code	The International Classification of Diseases, 9th Revision code.
