# Supplementary material for: Genome-wide meta-analysis of myasthenia gravis uncovers new loci and provides insights into polygenic prediction
Source: Nat Commun. 2024 Nov 13;15:9839. doi: 10.1038/s41467-024-53595-6 (PMC11560923; doi:10.1038/s41467-024-53595-6)
Supplement: Supplementary file 5 — Reporting Summary [file 41467_2024_53595_MOESM5_ESM.pdf]

nature portfolio

Corresponding author(s): Stephan Ripke

Last updated by author(s): 2023/10/05

Reporting Summary

Nature Portfolio wishes to improve the reproducibility of the work that we publish. This form provides structure for consistency and transparency in reporting. For further information on Nature Portfolio policies, see our Editorial Policies and the Editorial Policy Checklist.

Statistics

For all statistical analyses, confirm that the following items are present in the figure legend, table legend, main text, or Methods section.

n/a	Confirmed	
☐	☒	The exact sample size (*n*) for each experimental group/condition, given as a discrete number and unit of measurement
☐	☒	A statement on whether measurements were taken from distinct samples or whether the same sample was measured repeatedly
☐	☒	The statistical test(s) used AND whether they are one- or two-sided<br>*Only common tests should be described solely by name; describe more complex techniques in the Methods section.*
☐	☒	A description of all covariates tested
☐	☒	A description of any assumptions or corrections, such as tests of normality and adjustment for multiple comparisons
☐	☒	A full description of the statistical parameters including central tendency (e.g. means) or other basic estimates (e.g. regression coefficient) AND variation (e.g. standard deviation) or associated estimates of uncertainty (e.g. confidence intervals)
☐	☒	For null hypothesis testing, the test statistic (e.g. *F*, *t*, *r*) with confidence intervals, effect sizes, degrees of freedom and *P* value noted<br>*Give P values as exact values whenever suitable.*
☒	☐	For Bayesian analysis, information on the choice of priors and Markov chain Monte Carlo settings
☒	☐	For hierarchical and complex designs, identification of the appropriate level for tests and full reporting of outcomes
☐	☒	Estimates of effect sizes (e.g. Cohen's *d*, Pearson's *r*), indicating how they were calculated

Our web collection on statistics for biologists contains articles on many of the points above.

Software and code

Policy information about availability of computer code

Data collection	No custom code or software was used to collect the data

Data analysis	BEAGLE 5.4: [http://faculty.washington.edu/browning/beagle/beagle.html]<br>EIGENSTRAT: [https://alkesgroup.broadinstitute.org/EIGENSOFT/]<br>FUMA 1.5.2: [https://github.com/Kyoko-wtnb/FUMA-webapp]<br>FUSION: [http://gusevlab.org/projects/fusion/]<br>GCTA: [https://yanglab.westlake.edu.cn/software/gcta/]<br>RICOPILI:[https://sites.google.com/a/broadinstitute.org/ricopili/], [https://github.com/Ripkelab/ricopili]<br>LDSC: [https://github.com/bulik/ldsc]<br>IMPUTE C4: [https://github.com/freeseek/imputec4]<br>METAL: [http://www.sph.umich.edu/csg/abecasis/metal/]<br>EAGLE 2.4.1: [https://alkesgroup.broadinstitute.org/Eagle/]<br>MINIMAC 3: [http://genome.sph.umich.edu/wiki/minimac3]<br>PLINK 1.9: [http://pngu.mgh.harvard.edu/purcell/plink/]<br>PLINK 2.0 [https://www.cog-genomics.org/plink/2.0/]<br>SHAPEIT2: [https://jmarchini.org/software/#shapeit-2]<br>IMPUTE4: [https://jmarchini.org/software/#impute-4]

For manuscripts utilizing custom algorithms or software that are central to the research but not yet described in published literature, software must be made available to editors and reviewers. We strongly encourage code deposition in a community repository (e.g. GitHub). See the Nature Portfolio guidelines for submitting code & software for further information.

Data

Policy information about availability of data

All manuscripts must include a data availability statement. This statement should provide the following information, where applicable:

- Accession codes, unique identifiers, or web links for publicly available datasets
- A description of any restrictions on data availability
- For clinical datasets or third party data, please ensure that the statement adheres to our policy

Full summary statistics for the presented association analyses of MG, EOMG and LOMG will be made available via GWAS Catalog [https://www.ebi.ac.uk/gwas/downloads/summary-statistics].
1000 Genomes HLA reference: [https://github.com/WansonChoi/CookHLA/tree/master/1000G_REF].
The Chia et al. 2022 summary statistics (GWAS Catalog accession code GCST90093061): [https://www.ebi.ac.uk/gwas/studies/GCST90093061]
LDSC formatted summary statistics: [https://alkesgroup.broadinstitute.org/sumstats_formatted/]
Haplotype Reference Consortium reference panel release 1.1: [https://ega-archive.org/studies/EGAS00001001710]
Renton et al. 2015 MG cases (dbGAP accession code phs000726): [https://www.ncbi.nlm.nih.gov/projects/gap/cgi-bin/study.cgi?study_id=phs000726.v1.p1]
Controls merged with Renton et al., 2015 MG cases (dbGAP accession code phs000196): [https://www.ncbi.nlm.nih.gov/projects/gap/cgi-bin/study.cgi?study_id=phs000196.v3.p1]
Controls merged with Gregersen et al., 2012 EOMG cases (dbGAP accession code phs000882): [https://www.ncbi.nlm.nih.gov/projects/gap/cgi-bin/study.cgi?study_id=phs000882.v1.p1]

Research involving human participants, their data, or biological material

Policy information about studies with human participants or human data. See also policy information about sex, gender (identity/presentation), and sexual orientation and race, ethnicity and racism.

Reporting on sex and gender	We did not perform sex or gender based analyses.
Reporting on race, ethnicity, or other socially relevant groupings	We controlled for confounding stratification with exclusion via (1) principal component analysis outlier exclusions and (2) inclusion of principal component covariates. The study was limited to individuals of European ancestry.
Population characteristics	See above, reporting on race, ethnicity. Additionally, sub-analyses were conducted for age of onset and antibody status of myasthenia gravis cases.
Recruitment	Cases were recruited through clinical studies, population based registers through electronic healthcare records and by self-report in the replication sample (23andMe, Inc.).
Ethics oversight	The study presented was approved by the institutional review board of the Charite Universitaetsmedizin Berlin. The meta-analyzed studies' IRB approval information is included in the supplementary information.

Note that full information on the approval of the study protocol must also be provided in the manuscript.

Field-specific reporting

Please select the one below that is the best fit for your research. If you are not sure, read the appropriate sections before making your selection.

☒ Life sciences ☐ Behavioural & social sciences ☐ Ecological, evolutionary & environmental sciences

For a reference copy of the document with all sections, see nature.com/documents/nr-reporting-summary-flat.pdf

Life sciences study design

All studies must disclose on these points even when the disclosure is negative.

Sample size	Sample size was calculated based on all individuals with available phenotypic information on case and control status that were successfully genotyped, passed genomic quality control and imputation. 5,708 cases and 432,028 controls were included in the discovery sample, which is a 3-fold increase from previous publications.
Data exclusions	Data were excluded based on ancestry and poor genotyping quality.
Replication	The results were replicated in a sample of of 3,989 cases and 226,643 controls provided by 23andMe,Inc.
Randomization	Not applicable.
Blinding	Not applicable.

Reporting for specific materials, systems and methods

We require information from authors about some types of materials, experimental systems and methods used in many studies. Here, indicate whether each material, system or method listed is relevant to your study. If you are not sure if a list item applies to your research, read the appropriate section before selecting a response.

Materials & experimental systems

n/a	Involved in the study	
☒	☐	Antibodies
☒	☐	Eukaryotic cell lines
☒	☐	Palaeontology and archaeology
☒	☐	Animals and other organisms
☒	☐	Clinical data
☒	☐	Dual use research of concern
☒	☐	Plants

Methods

n/a	Involved in the study	
☒	☐	ChIP-seq
☒	☐	Flow cytometry
☒	☐	MRI-based neuroimaging
